# A Multimodal Neuroprosthetic Interface to Record, Modulate and Classify Electrophysiological Biomarkers Relevant to Neuropsychiatric Disorders

**DOI:** 10.3389/fbioe.2021.770274

**Published:** 2021-11-03

**Authors:** Bettina Habelt, Christopher Wirth, Dzmitry Afanasenkau, Lyudmila Mihaylova, Christine Winter, Mahnaz Arvaneh, Ivan R. Minev, Nadine Bernhardt

**Affiliations:** ^1^ Department of Psychiatry and Psychotherapy, Medical Faculty Carl Gustav Carus, Technische Universität Dresden, Dresden, Germany; ^2^ Leibniz Institute of Polymer Research Dresden, Dresden, Germany; ^3^ Department of Automatic Control and Systems Engineering, University of Sheffield, Sheffield, United Kingdom; ^4^ Biotechnology Center (BIOTEC), Center for Molecular and Cellular Bioengineering (CMCB), Technische Universität Dresden, Dresden, Germany; ^5^ Department of Psychiatry and Psychotherapy, Charite University Medicine Berlin, Campus Mitte, Berlin, Germany

**Keywords:** event-related potentials/ERP, brain stimulation, neuroprosthetics, flexible electronics, biomedical interfaces, machine learning, neuropsychiatric disorders

## Abstract

Most mental disorders, such as addictive diseases or schizophrenia, are characterized by impaired cognitive function and behavior control originating from disturbances within prefrontal neural networks. Their often chronic reoccurring nature and the lack of efficient therapies necessitate the development of new treatment strategies. Brain-computer interfaces, equipped with multiple sensing and stimulation abilities, offer a new toolbox whose suitability for diagnosis and therapy of mental disorders has not yet been explored. This study, therefore, aimed to develop a biocompatible and multimodal neuroprosthesis to measure and modulate prefrontal neurophysiological features of neuropsychiatric symptoms. We used a 3D-printing technology to rapidly prototype customized bioelectronic implants through robot-controlled deposition of soft silicones and a conductive platinum ink. We implanted the device epidurally above the medial prefrontal cortex of rats and obtained auditory event-related brain potentials in treatment-naïve animals, after alcohol administration and following neuromodulation through implant-driven electrical brain stimulation and cortical delivery of the anti-relapse medication naltrexone. Towards smart neuroprosthetic interfaces, we furthermore developed machine learning algorithms to autonomously classify treatment effects within the neural recordings. The neuroprosthesis successfully captured neural activity patterns reflecting intact stimulus processing and alcohol-induced neural depression. Moreover, implant-driven electrical and pharmacological stimulation enabled successful enhancement of neural activity. A machine learning approach based on stepwise linear discriminant analysis was able to deal with sparsity in the data and distinguished treatments with high accuracy. Our work demonstrates the feasibility of multimodal bioelectronic systems to monitor, modulate and identify healthy and affected brain states with potential use in a personalized and optimized therapy of neuropsychiatric disorders.

## Introduction

The prefrontal cortex (PFC) represents a fundamental structure for top-down behavioral control and modulates attention, working memory, reward evaluation and the ability to self-control actions, emotions and stress. Disturbances of prefrontal neural network activity underlie many cognitive and behavioral impairments observed in neuropsychiatric diseases such as addictive disorders, schizophrenia or autism. The complex etiology of these diseases makes optimal treatment challenging (Gao et al., 2012). Current therapeutical interventions often present with side effects and lack long-term efficacy. In particular, systemic pharmacotherapy shows poor topical specificity and adaptability to changes in patient-specific needs regarding treatment duration and intensity (Olofsson and Bouton, 2019). The chronic reoccurrence and high relapse rates following treatment (Agenagnew and Kassaw, 2020) thus warrant new approaches.

Brain stimulation techniques, such as Deep Brain Stimulation (DBS) and transcranial Direct Current Stimulation (tDCS), have gained increasing attention as alternative treatment options. Predominantly applied in Parkinson’s disease to improve motor function (Limousin and Foltynie, 2019), DBS also showed beneficial effects in neuropsychiatric disorders such as depression (Merkl et al., 2013), drug addiction (Kuhn et al., 2011; Ge et al., 2019), as well as obsessive-compulsive and anxiety disorders (Sturm et al., 2003). However, due to its invasiveness and continuous stimulation mode, DBS holds the risk of side effects such as impaired speech, gait and cognition (Buhmann et al., 2017) and has therefore been restricted to a small number of severe and otherwise treatment-resistant cases. In contrast, tDCS offers scalp-applied and thus non-invasive brain stimulation with no or just minimal side effects (Bastani and Jaberzadeh, 2012). Prefrontal tDCS has been shown to reduce symptoms of depression (Boggio et al., 2008) and schizophrenia (Gomes et al., 2018), as well as craving and drug consumption in substance use disorders (Song et al., 2019). However, reports of varying treatment efficacy (Herrera-Melendez et al., 2020) in response to tDCS might be due to identical stimulation parameters used for all subjects in a rigid “one-size-fits-all” fashion not taking into account individual differences in brain anatomy, underlying pathology and temporal changes in brain states (Karabanov et al., 2016). Furthermore, up to ∼75% of epicranially applied currents are attenuated by scalp and skull (Vöröslakos et al., 2018) hampering target region and dose specification.

Epicortical neuroprosthetics, equipped with multiple sensing and stimulation modalities, might offer a new off-the-beaten-track toolbox for diagnosis and therapy and may overcome some of the limitations of current brain stimulation techniques. Implanted epi- or subdurally and made of soft and biocompatible materials (Minev et al., 2015; Athanasiadis et al., 2019; Afanasenkau et al., 2020), epicortical devices can adapt to the brain’s curvilinear surface, resulting in reduced tissue response and improved long-term stability compared to brain penetrating electrodes (Leuthardt et al., 2006; Degenhart et al., 2016). Furthermore, direct cortical stimulation via small surface electrodes provides effective and precise stimulation close to the target structure (Caldwell et al., 2019). Moreover, epicortical neuroprosthesis enables combined neuromonitoring and stimulation in one device, allowing immediate detection of stimulation effects on neural activity. Current clinical applications of epicortical electrodes for electrocorticography (ECoG) and direct cortical stimulation focus on real-time functional brain mapping to assess language, motor and sensory function during surgical intervention for medically intractable epilepsy and brain tumors (Caldwell et al., 2019; Mahon et al., 2019). Besides intraoperative epileptic seizure localization, an ECoG-type array combined with direct cortical stimulation has been successfully implemented to reduce an incipient seizure by detecting abnormal neural activity that subsequently triggers stimulation. This so-called Responsive Neurostimulation System® (NeuroPace®, Mountain View, CA, United States ) is the first demonstration of a genuinely bidirectional, closed-loop brain-computer interface approved for clinical application (Sun and Morrell, 2014; Caldwell et al., 2019).

Closed-loop neuromodulation may also open opportunities to treat neuropsychiatric disorders by utilizing artificial intelligence and advanced machine learning algorithms to identify brain states and optimize stimulation parameters based on pre-defined neural features (Fellous et al., 2019). Event-related potentials (ERPs) present eligible biomarkers for this purpose. These short electrical deflections induced in the brain immediately following an external or internal event have proven valuable for investigating sensory information processing and higher-order cognition in healthy individuals as well as psychopathological conditions (Sokhadze et al., 2017). Within scalp-recorded electroencephalograms (EEG), ERPs appear as time-locked local negative or positive maxima of a few microvolts (µV) lasting tens to hundreds of milliseconds (ms). They are commonly labelled according to their polarity (negative = N, positive = P) and latency (in ms post-stimulus or order of appearance within the recorded waveform) (Kappenman and Luck, 2011). The best-known paradigm to elicit ERPs is the” oddball “paradigm in which subjects are confronted with a series of frequent (e.g., auditory or visual) stimuli (“standards”) randomly interspersed with rare stimuli (“deviants”). Standard ERP components observed in such tasks include P1, N1, P2, N2 and P3. The components P1, N1 and P2 reflect pre- and early attentive automatic stimulus processing and sensory gating that constitutes an inhibitory filter mechanism to focus on salient stimuli while disregarding irrelevant or repetitive information (Lijffijt et al., 2009). The N2 is elicited by rare events and reflects a change-detection response sensitive to novelty and stimulus probability (Folstein and Van Petten, 2008; Campanella et al., 2014). Likewise, amplitudes of the P3 vary with stimulus incidence and significance but also depend on a subject’s motivation, attentional resources and cognitive capabilities (Polich, 2007; Campanella et al., 2014). Modified ERPs indicate impaired PFC functioning and cognitive deficits associated with neuropsychiatric diseases (Knight et al., 1999). For example, delayed and/or reduced ERP amplitudes have been observed in alcohol-addicted patients and animal models (Ehlers, 1988; Cohen et al., 2002; Marco et al., 2005; Ehlers et al., 2014). Primarily a disturbed P3 component has been related to poor behavior control and increased relapse probability and therefore judged as a suitable predictor for the relapse risk after drug withdrawal (Petit et al., 2015; Luijten et al., 2016).

ERPs measured by scalp-EEG have high temporal precision but lack spatial resolution, are sensitive to noise, and, like in tDCS, electrical signals are partly silenced through the skull (Voss and Paller, 2017). However, ECoG electrodes are closer to the source of relevant brain activity and have demonstrated superior signal sensitivity, broader bandwidth, higher topographical resolution and a lower vulnerability to artifacts than EEG, resulting in accurate ERP acquisition (Leuthardt et al., 2006; Krusienski and Shih, 2010).

So far, ECoG electrodes have been placed over lateral, sensorimotor areas based on clinical requirements for epileptic seizure localization (Krusienski and Shih, 2010; Mouthaan et al., 2016) or to enable paralyzed patients to control external devices using movement-related neural activity patterns (Vansteensel et al., 2016; Benabid et al., 2019). However, the potential of an epicortical implant to target cognitive ERPs from central prefrontal brain regions remains unexplored.

Based on the advantages and opportunities of an implanted bidirectional brain-computer interface, we set out to build a tailor-made soft and multimodal epicortical device able to both measure and modulate neurophysiological features relevant to the diagnosis and therapy of neuropsychiatric disorders. Modulation can hereby be achieved through implant-driven electrical stimulation or local drug application. We implanted our device epidurally above the medial (m) PFC of rats and tested its feasibility to obtain auditory ERPs. We detected a reduction in neuronal activity induced by systemic acute alcohol intake. Moreover, implant-driven neuromodulation was observed following direct cortical application of electrical pulses and pharmacologically active naltrexone (NTX). We furthermore deployed machine learning algorithms to distinguish treatment-specific brain responses from single ERP trials with potential use as feedback for closed-loop adjustment of neurostimulation in a therapeutic neuroprosthesis. Finally, we performed an immunohistochemical analysis of implant and intervention tolerability.

## Materials and Methods

### Design and Fabrication of the Neuroprosthetic Device

We initially developed a custom implantable device covering the surface of the frontal lobe of the rat cortex. The neuroprosthetic devices were manufactured using the 3D bioprinter 3DDiscovery^TM^ Evolution (regenHU Ltd., Villaz-St-Pierre, Switzerland). The design of the implants was prepared with the printers’ bioprinting software suite BioCAD^TM^ and in G-code using a custom-developed Python (Version 3.7) based software. Implants consisted of electrodes arranged in a 3 × 3 matrix with a distance between adjacent electrodes of 1.5 mm in the mediolateral direction and 2.0 mm in the rostrocaudal direction. According to their position above the mPFC, electrodes were labelled as frontocentral (FC), frontal left (FL), frontal right (FR), medial central (MC), medial left (ML), medial right (MR), posterior central (PC), posterior left (PL) and posterior right (PR). Eight of the electrodes (0.2 × 0.2 mm^2^) were used for neural recording only. The larger FC electrode (1 × 1 mm^2^) was used for both, recording and electrical stimulation of the mPFC spanning both hemispheres at 3.2 mm anterior to bregma. In addition, a microfluidic channel was integrated to enable local delivery of liquids at 2.2 mm anterior to bregma.

**FIGURE 1:**
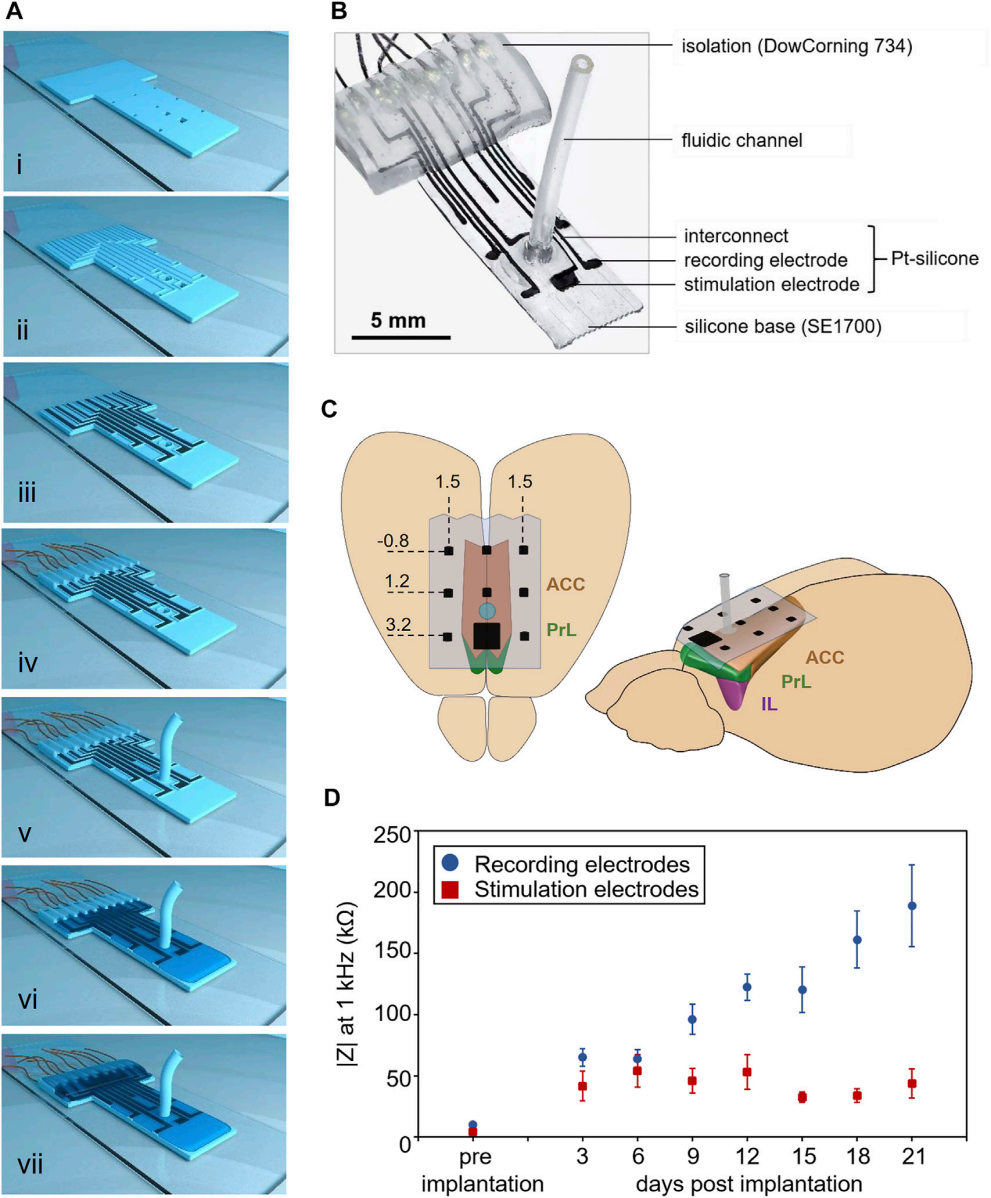
Multimodal epicortical array for recording and modulation of neural activity. **(A)** i) Silicone base layer (DOWSIL™ SE1700), including holes defining the active sites of electrodes and the microfluidic channel. ii) A second silicone layer defines the borders for electrode interconnects and the microfluidic channel. iii) The conductive portions of the array are formed from a platinum-silicone composite. iv) Connection of microwires. v) Connection of silicone fluidic channel. vi) Isolation of the array using SYLGARD^TM^184. vii) Isolation of the cable contacts using DOW CORNING® 734. **(B)** Photograph of an implant **(C)** Implantation of the array above the rat mPFC encompassing anterior cingulate (ACC), prelimbic (PrL) and infralimbic (IL) cortices. Stereotactic coordinates (mm) relative to bregma. **(D)** Impedances *in vitro* (at 1 kHz) of recording (blue, n = 80 electrodes of 10 implants) and stimulation electrodes (red, n = 10 electrodes of 10 implants) and *in vivo* (varying implant numbers, see Supplementary Table S2). Data are presented as mean ± SEM.

Arrays were 3D-printed layer-by-layer on glass slides previously treated with a release layer (2% sodium dodecyl sulfate). The implants were assembled with a 100 µm thick base layer of a silicone elastomer (DOWSIL™ SE 1700, Dow Inc., Midland, United States ) with interrupts defining the position of the active sites of electrodes and the outlet of the microfluidic channel (Figures 1Ai). Subsequently, borders, determining the interconnect paths, were printed using the same material (Figure 1Aii). Next, we deposited electrically conductive tracks consisting of platinum powder (chemPUR, Karlsruhe, Germany) dispersed in tri(ethylene glycol) monomethyl ether (TGME, Merck KGaA, Darmstadt, Germany) (Figure 1Aiii). The quality of all printing and processing steps was evaluated under a microscope (Supplementary Figure S1), and defects were manually corrected. After printing each layer, the implants were placed on a heating plate at 120°C to enable polymerization and solvent evaporation. Then, a drop of polydimethylsiloxane (PDMS, SYLGARD^TM^184, Dow Inc., Midland, United States) pre-cured for 90 s at 90°C was manually applied at the position of each electrode and baked on a hot plate at 105°C. The application and baking were made for each electrode separately to ensure rapid polymerization and prevent a PDMS film formation covering the electrode site. To interface the implant with external electronic devices, stainless steel microwires (∅: 0.23 mm, 7SS-2T, Science Products GmbH, Hofheim, Germany) were manually attached to the interconnects with a silver-containing epoxy adhesive (EPO-TEK® H27D, part A, Epoxy Technology Inc., Billerica, United States) (Figure 1Aiv). The solvent was then evaporated at 90°C. For the microfluidic channel, a section of soft silicone tubing (length: 1 cm, inner ∅: 0.51 mm, 45630102, DowCorning Silastic, Freudenberg Medical Europe GmbH, Kaiserslautern, Germany) was manually connected to the implant with SE1700 silicone (Figures 1AV). Implants were insulated with a PDMS layer polymerized at 90°C (Figure 1Avi). The wire connection was insulated with a thick silicone layer using DOW CORNING®734 (Figure 1Avii). Finally, the free endings of the microwires were soldiered to an off-the-shelf connector (TC-2506280, Conrad Electronic SE, Hirschau, Germany) and insulated with hot glue. A finished electrode array is presented in Figure 1B.

### Electrochemical Impedance Measurements

The impedances of implant electrodes *in vitro* were recorded in phosphate buffered saline (PBS) (pH 7.4) at room temperature using a potentiostat equipped with a frequency response analyzer (AUTOLAB PGSTAT204, Deutsche Metrohm Prozessanalytik GmbH and Co., KG, Filderstadt, Germany). A platinum wire served as a counter electrode and an Ag/AgCl electrode as reference. Impedance measurements *in vivo* were carried out right before each recording session using the Intan RHD2000 USB interface system (Intan Technologies, Los Angeles, United States). The counter electrode was a stainless steel wire (7SS-2T, Science Products GmbH, Hofheim, Germany) whose de-insulated ending was connected to a microscrew fixed into the interparietal bone.

### Animals

All investigations within this project have been approved by the ethics committees of the Technical University of Dresden and the Saxonian ministry of the interior (Landesdirektion Sachsen, TVV 58/2018). Experiments were performed according to the guidelines of the Directive 2010/63/EU on the protection of animals used for scientific purposes of the European Commission with great attention to avoid suffering and reduce the number of animals used.

The study involved n = 10 adult male Wistar wildtype rats (Janvier Labs, Le Genest-Saint-Isle, France) initially housed in groups of up to four animals. After surgery, rats were housed in single cages (Makrolon®, Type III, Tecniplast Deutschland GmbH, Hohenpeißenberg, Germany) on sawdust bedding (Ssniff - Bedding 3/4 S, Altrogge, Lage, Germany) and with Bed-r'Nest material (Datesand Ltd., Bredbury, United Kingdom) as enrichment. Pelleted food (V1534-300, ssniff Spezialdiäten GmbH, Soest, Germany) and water were available ad libitum. Housing rooms were temperature (20–22°C) and humidity (50–60%) controlled with a 12 h automatic light-dark cycle with lights on at 6.00 am. Before surgery, animals were habituated to the experimenter and the recording set-up through daily handling for 2 weeks.

### Implantation of the Neuroprosthesis

Surgeries to implant the bioelectronic devices were performed under subcutaneous anesthesia with fentanyl (0.005 mg/kg), midazolam (2.00 mg/kg), and medetomidinhydrochloride (0.135 mg/kg) injected into a nuchal fold. An animal’s head was fixed into a stereotactic frame via ear pins and jaw brackets. First, the skullcap and cranial suture were exposed and two microscrews were drilled into the skull: the first one in the left parietal skull bone, which was later cable-connected to the implant connector and served as a reference and the second one in the right frontal skull bone serving as anchor screw to improve fixation. Then, the skull was slowly trepaned (∅ 6.0 mm, <1,500 rpm) under constant flushing with cold PBS at −2.6–3.2 mm bregma. The dura mater was then carefully incised bilaterally next to the position of the microchannel outlet (2.2 mm anterior to bregma) to allow the influx of NTX solution. The implant was placed centrally on the cortex with the stimulation electrode located at 3.2 mm anterior to bregma (Figure 1C). Synthetic dural sealant (1A:3B, 3–4,680, Dow Corning, Midland, United States) was applied to the implant to close the drill hole. The external parts of the implant were fixed to the skull with dental cement (Paladur, Kulzer GmbH, Hanau, Germany), and the wound was sutured. Upon completion of surgery, anesthesia was antagonized by subcutaneous injection of naloxon (0.12 mg/kg BW), flumazenil (0.2 mg/kg) and atipamezole (0.75 mg/kg). Animals received meloxicam (1.0 mg/kg, s.c.) as pain medication right after surgery and on the following day.

### ECoG Recording and Stimulation Set-Up

**FIGURE 2:**
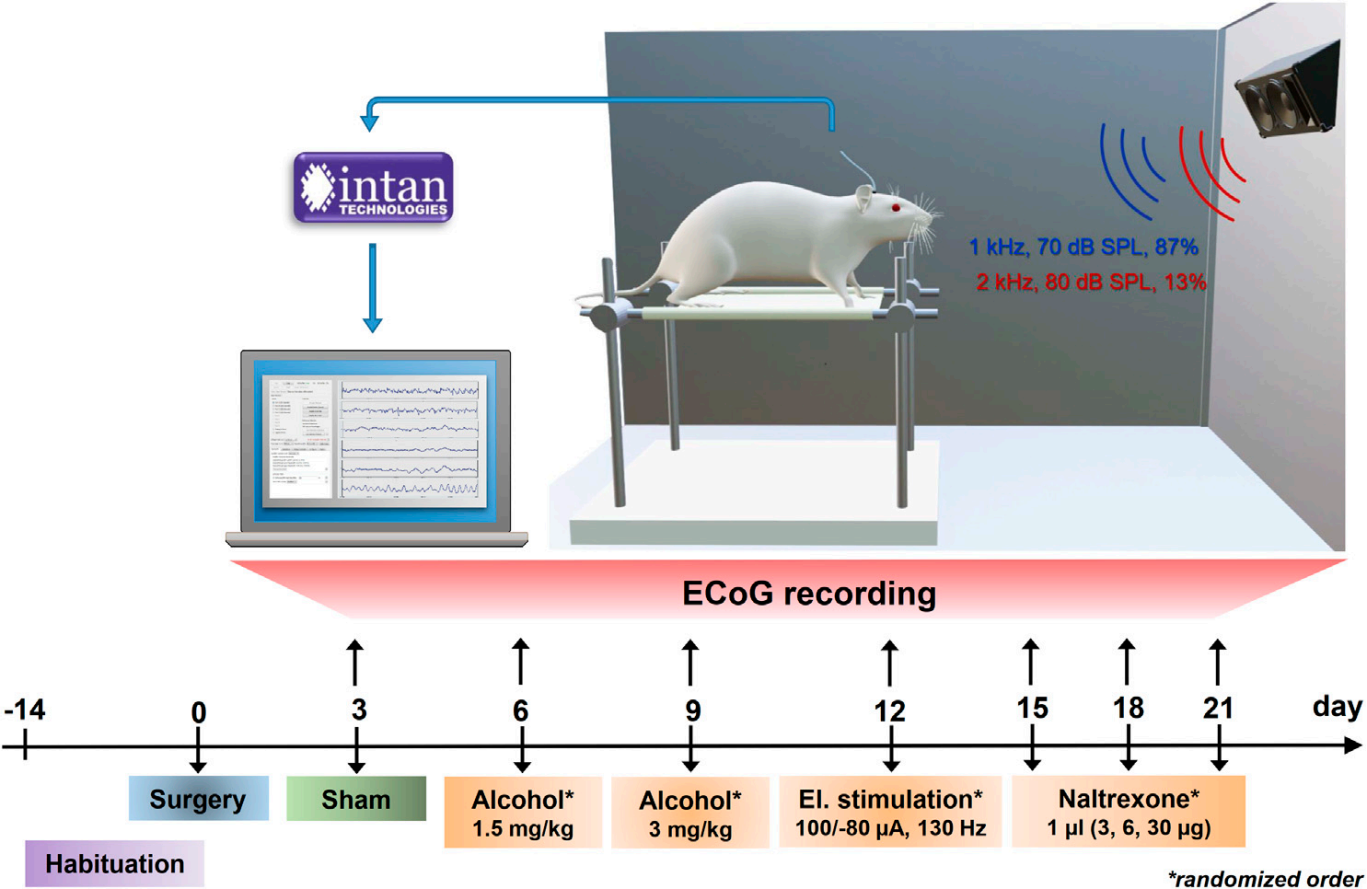
Set-up and timeline for ECoG recording sessions. Experiments were planned as a within-subjects-design with all animals undergoing an initial habituation period, surgical intervention and neural recordings without previous treatment (sham) and following alcohol injections, electrical brain stimulation and cortical administrations of NTX in a randomized order. Electrical potentials elicited during the auditory oddball task were amplified and digitized at a sampling rate of 3 kHz via the Intan RHD2000 recording system and visualized and saved on a computer using the Intan recording software.

ECoG recordings started 3 days after surgery and were performed at a sampling rate of 3 kHz using the Intan RHD2000 USB interface system with the RHD2132 amplifier chip (Intan Technologies, Los Angeles, United States), cable-connected to the implant connector. Recordings were initially performed without and after that—in randomized order—every 3 days following intraperitoneal injection of 1.5 or 3 g/kg EtOH (20% v/v, 20 min before recording), electrical or pharmacological stimulation with NTX (Figure 2). Electrical stimulation of the mPFC through the frontocentral electrode was applied as biphasic, charge-imbalanced pulses (100 µA anodal/-80 µA cathodal, 130 Hz) for 20 min before recording using a computer-interfaced current generator (STG4004, MultiChannelSystems, Reutlingen, Germany). For pharmacological stimulation, NTX (Merck KGaA, Darmstadt, Germany) was dissolved in artificial cerebrospinal fluid (125 mM NaCl, 3 mM KCl, 2.5 mM CaCl_2_, 1.3 mM MgSO_4_, 1.25 mM NaH_2_PO_4_, 26 mM NaHCO_3_, 13 mM C_6_H_12_O_6_) and applied in different dosages (3, 6 or 30 μg/μl) at a volume of 1 µl via the implant’s microchannel 20 min before recording.

Auditory stimuli to induce ERPs were generated using custom-written Matlab scripts (Version R2019a, The Mathworks Inc., Natick, MA, United States) and consisted of frequent (standards: 50 ms, 1 kHz, 70 dB SPL, 1,200 times = 87% of trials) and rare (deviants: 50 ms, 2 kHz, 80 dB SPL, 180 times = 13% of trials) sinusoidal sounds with rise and fall times of 5 ms and with 1 s interstimulus interval. Deviant tones were interspersed with at least one standard sound avoiding that two deviants occurred successively. To reduce movement artefacts, one animal at a time was placed in a rodent sling (Lomir Biomedical Inc., Notre-Dame-de-l'Île-Perrot, Canada) within an electrically shielded and soundproofed audiometry booth. Sound stimuli were presented in 5 blocks of 5 min via loudspeakers at a distance of 40 cm and an angle of 45° centrally above the animals’ head (Figure 2).

### Data Processing and Analysis

Data processing was performed using the EEGLAB toolbox (Delorme and Makeig, 2004) (Version 2019.1) for Matlab. Initially, data were filtered offline using a 0.1–45 Hz bandpass FIR filter (Kaiser windowed, Kaiser *β* = 5.65, filter length 54,330 points). Data were segmented in epochs between −100 and 700 ms relative to stimulus onset separately for standard and deviant sounds and baseline-corrected using these epoch’s pre-stimulus interval (−100–0 ms). Artifacts were identified and excluded based on a delta criterion of 400 µV before averaging epochs for single subjects and all animals (grand average), respectively. ERP peak latencies were identified within standard time intervals confirmed by visual inspection (P1: 30–75 ms, N1: 80–105 ms, P2: 110–125 ms, N2: 130–180 ms, P3: 200–500 ms). The amplitudes of the ERP components were calculated as averaged amplitudes within a time window of 10 ms around the peak latency. Amplitudes of the P2 component are N1-P2 peak-to-peak amplitudes, whereas all other components are baseline-to-peak amplitudes.

### ERP Single-Trial Classification

Filtered and artifact-free neural recording files already segmented for standard and deviant sounds were used for single-trial ERP classification. In addition, procedures were performed on the data provided by the frontocentral electrode, as this was the channel in which successful recordings were consistently available, only missing NTX3 for one animal.

### Dataset Generation for Treatment Classification

Data were low pass filtered using a least squares linear phase anti-aliasing FIR filter with a cut-off frequency of 32 Hz and downsampled to 64 Hz to reduce dimensionality and the possibility of overfitting. Time domain data were then extracted in the ranges of the N1 (80–105 ms) and P3 (200–500 ms), resulting in a total number of 22 feature time points per trial. Next, ERP “difference trials” for individual sessions and animals were generated by subtracting 1) the mean response to standard stimuli from each response to a deviant stimulus and 2) each response to a standard stimulus from the mean response to deviant stimuli. Applying both of these approaches allows a more extensive training set than performing only one of the methods. Finally, all difference trials from all other animals were combined to form the training data set. Difference trials from an animal undergoing the classification procedure were excluded from the training set.

### Feature Selection and Classification

Next, we performed a stepwise linear discriminant analysis (SWLDA), chosen as a combined feature selection and classification strategy. SWLDA has previously been proven effective when processing ERP data (Krusienski et al., 2006), including performing single-trial classification (Wirth et al., 2020). Time-domain features (i.e., the voltage values recorded at each time point, after pre-processing) were used for the feature selection phase. SWLDA feature selection first involved creating an initial model with no features and subsequent stepwise regression performed on the training data. Regression analysis was performed on potential models during each step, including or excluding each feature in turn and producing a F-statistic with a *p*-value for each feature. Smaller *p*-values indicated features with the highest likelihood of being beneficial. If any feature not already in the model had a *p*-value below the entry threshold of 0.05, the feature with the smallest *p*-value would be added to the model. If no features were added, but any feature currently in the model now had a *p*-value above the removal threshold of 0.1, then the feature with the highest *p*-value would be removed from the model. For example, upon starting the feature selection process for a given dataset, new models would be generated containing each individual time-domain feature, and the performance of each of these models on the training data would be compared to the performance of the empty set. If we imagine that a feature achieved the lowest *p*-value at t = 250 ms, and that this *p*-value was below 0.05, the model containing only this feature would be the current model at the end of the first step. During the second step, models containing each other available feature, together with the feature at t = 250 ms, would be generated, and their performance on the training data would be compared to that of the current model. If the lowest *p*-value was achieved by a feature at t = 90 ms, and this *p*-value was below 0.05, the current model at the end of the second step would be the model containing both features at t = 90 ms and t = 250 ms. These steps continued until no features were added to, or removed from, the model. If this process failed to select any features, the single feature with the smallest *p*-value would be selected. For the classification phase, a linear discriminant analysis model was trained and tested using the selected features. Each training trial is represented as a point in a n-dimensional space to build the model, where n is the number of selected features. A linear hyperplane is then fitted in this n-dimensional space to separate best the two sets of points representing the two classes. The class with the fewest training trials was oversampled to ensure that training occurred with an equal number of trials per class. Using this method, each difference trial in the test set from a given session was classified. In order to obtain these single-trial classifications, the test trial was represented as a point in the n-dimensional space. Depending on which side of the hyperplane it lay, it would be classified as either treatment. A simple majority vote was then carried out, based on the classifier outputs of all test trials in the session, to provide an overall session-level classification of which treatment had been applied. This approach was tested on one-vs-one combination for all interventions. For all treatment comparisons, each session’s actual treatments and predicted treatments were cross-tabulated to form a contingency table on which a chi-square test was performed. The classification of a given treatment comparison was considered statistically significant overall if the *p*-value of the chi-square statistic was less than 0.05.

### Immunohistochemistry

Immunohistochemical evaluation of implant and treatment biocompatibility were performed on three groups: 1) animals that received the surgical intervention but no implants (sham, n = 3), 2) rats with a non-functional implant (dummy, n = 3) and 3) animals with an active implant that received a combination of EtOH injections, electrical stimulation and NTX delivery (treatment, n = 7). After 4 weeks of implantation, rats were perfused with PBS and paraformaldehyde (PFA), and brains were extracted and stored at 4°C in PFA for 24 h. To dehydrate the tissue, brains were kept in sucrose (30%) at 4°C for up to 1 week before freezing them into methylbutan within liquid nitrogen at −40°C for 2 min. Brains were stored at −80°C until further processing. Brains were cut into slices of 40 µm thickness using a microtome and kept free-floating into antifreeze medium at −20°C until immunohistochemical staining. Staining was performed as double-staining. From each brain, six slices were used per double-staining. Each first slice was derived from underneath the stimulation electrode at 3.2 mm anterior to bregma. Consecutive sections were always 24 sections apart, covering the complete area underneath the implant. Slices were stained according to a standard staining protocol for free-floating sections. Each step preceded multiple washes in PBS. Sections were blocked in PBS containing 0.3% Triton X-100 and 10% serum for 2 h before incubation at 4°C overnight with the following primary antibodies in blocking solution (Supplementary Table S1): glial fibrillaric acidic protein (GFAP), ionized calcium binding adaptor molecule 1 (Iba1), laminins and platelet endothelial cell adhesion molecule (also known as cluster of differentiation 31 (CD31)), hexaribonucleotide binding protein-3 (NeuN) and cysteine-aspartic acid protease (caspase3). The following day, slices were incubated with secondary antibodies and fluorescent dye (Table S1) in blocking solution for 2 h before mounting on slides using Mowiol.

Fluorescent images of the brain sections were acquired with 10x magnification using the ZEISS AxioScan.Z1 Digital Slide Scanner. Image analysis was performed using the image processing suite Fiji (Schindelin et al., 2012). Thereby, images were initially background-corrected and a global threshold was applied to extract relevant objects (Supplementary Figure S2). We defined a region of interest (ROI) covering the entire implant width and all cortical layers up to a depth of 2 mm for each brain slice. The density of immunostaining (percentage of stained area per ROI, counts of stained objects per mm^2^) and mean fluorescence of the six brain slices per staining were averaged for each animal for statistical analysis.

### Statistical Analysis

Statistical analysis was carried out using SPSS® (Version 25, IBM Corp., Armonk, NY, United States). Initial ERP data of untreated animals underwent a one-sample *t*-test of the difference curve (deviant minus standard) against zero value. Treatment-induced modulations of neural activity were analyzed applying two-tailed paired *t*-tests (*α* = 0.05) for each treatment vs. sham condition. The resulting *p*-values were corrected post-hoc for multiple comparisons (accounting for false positives amongst the 9 channels) using the Benjamini-Hochberg procedure with a threshold of 5% and additionally reported as FDR-adjusted *p*-values. Effect sizes were calculated using Cohen’s *d* with differences of means divided by their standard deviation. Statistical analysis of immunohistochemical investigations involved a one-way analysis of variance and Holm-Sidak post-hoc testing comparing numbers of stained objects, percentage of total area stained and fluorescence intensities between sham-operated animals, rats receiving an implant dummy and treatment conditions. Effect sizes were calculated using Cohen’s *f*.

## Results

### Development of an Epicortical Neuroprosthesis to Interface the PFC

We used a recently established prototyping technology allowing rapid fabrication of soft and customized bioelectronic implants involving direct ink writing of silicones and a conductive platinum ink (Athanasiadis et al., 2019; Afanasenkau et al., 2020). Impedances of the soft electrodes at 1 kHz measured *in vitro* were 10.14 ± 1.96 kΩ (mean ± standard error of the mean (SEM)) for recording electrodes (n = 80 from 10 implants) and 4.36 ± 1.41 kΩ (n = 10 from 10 implants) for stimulating electrodes (Figure 1D; Supplementary Figure S3; Supplementary Table S2).

The bioelectronic devices were implanted epidurally above the mPFC in a delicate surgical procedure involving trepanation directly above the superior sagittal sinus and adjacent blood vessels. Attention was paid to limiting drilling to a few seconds at a low drill rotational speed under constant PBS flushing to prevent thermal tissue damage though occasional microbleedings could not be avoided.

The stability of implants *in vivo* was evaluated for up to 3 weeks, during which impedances of individual electrodes were measured every third day right before recordings. A general trend of impedance increase was observed over time in recording electrodes, while impedances of the stimulation electrodes remained stable (Figure 1D; Supplementary Table S2). Functional electrodes suitable for further ERP analysis were defined as having an impedance below 600 kΩ. Throughout the study period, more than 80% of electrodes remained functional (Supplementary Table S2). However, one of the stimulation electrodes lost functionality at the last session time point.

### Implementation of ECoG Measurements of PFC Activity

We set out to establish if our technology is capable to reliably obtain characteristic ERPs in awake rats (n = 10). We used an auditory oddball paradigm similar to those applied in human ERP studies to elicit typical sound-specific ERP responses.

**FIGURE 3:**
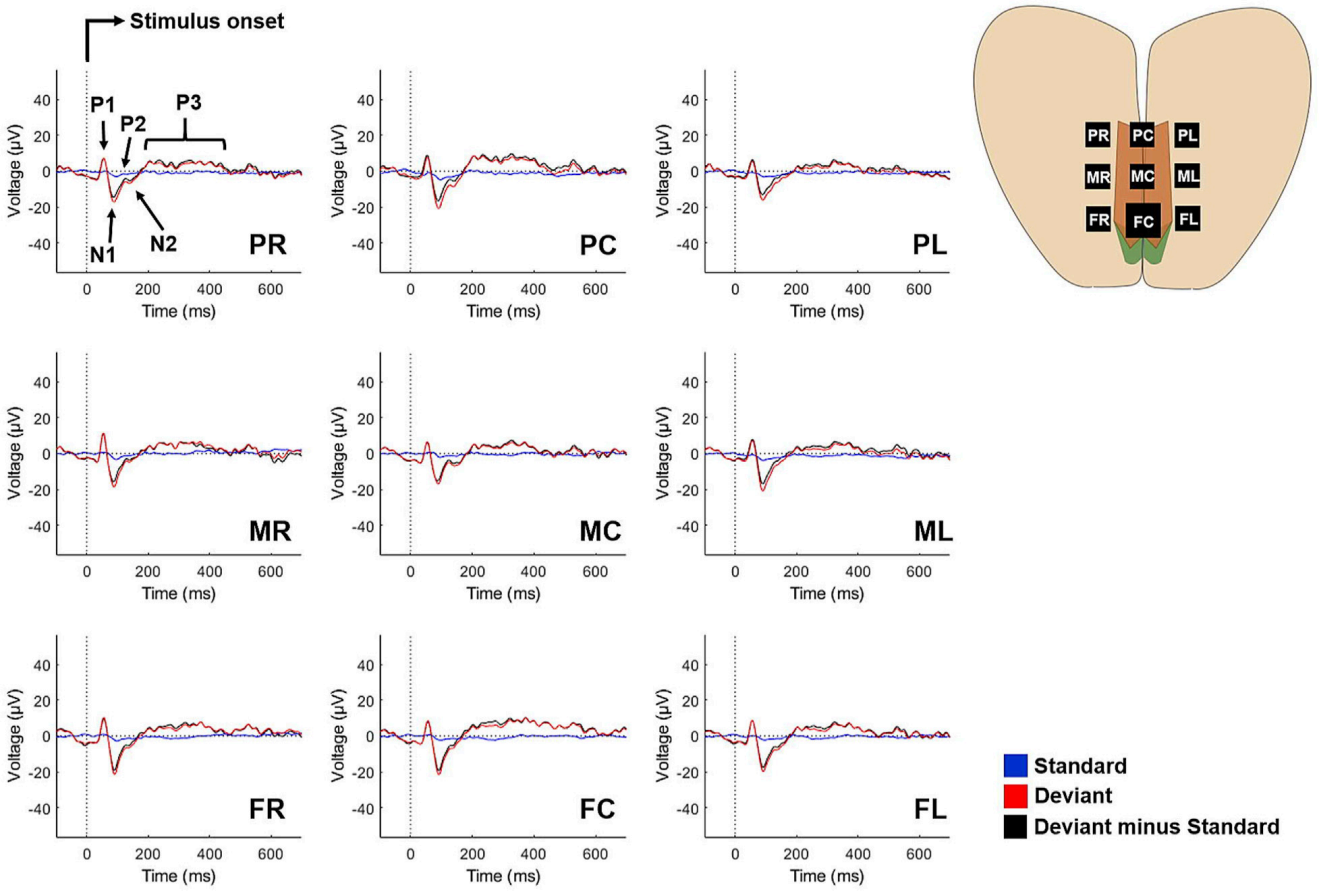
Grand average ERPs elicited by the standard sound (blue), deviant sound (red) and their difference curves (black) at all electrode sites. Traces are averaged from 10 animals. The components P1-N1-P2 are characteristic for pre- and early attentive auditory signal processing, while N2 and P3 indicate conscious stimulus evaluation.

Differences in neural activity underlying perception of the frequent standard and rare deviant sounds should result in different voltage waveforms recorded through the implant. Indeed, grand average ERPs over all animals revealed significantly different neural activities elicited by the two sounds with large effect sizes for P1, N1 and P3 components at all electrode sites, also bearing FDR correction (Figure 3; Supplementary Table S3). The components P2 and N2 were not clearly recognisable at each electrode though significant differences within these time intervals have been detected at some channels (e.g., electrode FL, P2: *t*(9) = 2.318, *p* = 0.046 uncorrected; N2: *t*(9) = -2.514, *p* = 0.033 uncorrected) but did not withstand FDR correction.

### Electrical and Pharmacological Modulation of Neural Activity

Following the successful acquisition of ERPs in untreated animals (sham), we tested if we can detect changes in neural activity induced by acute systemic alcohol administration, as well as electrical stimulation and NTX, both applied directly to the cortex through the implant. Following each of the interventions, animals were subjected to the same auditory oddball task. Unfortunately, interventions could not be performed on all animals due to issues with connectors, cement adhesion or blockage of the microfluidic channel, which necessitated these animals to be dropped from the study.

In each treatment condition, neural responses to the standard sound appeared flat, as was the case for untreated animals (presumably due to habituation effects caused by the high rate of repetitions). Therefore and in order to focus on treatment-induced changes of neural activity, subsequent analysis was performed on ERP difference curves (deviant-minus-standard sounds).

**FIGURE 4:**
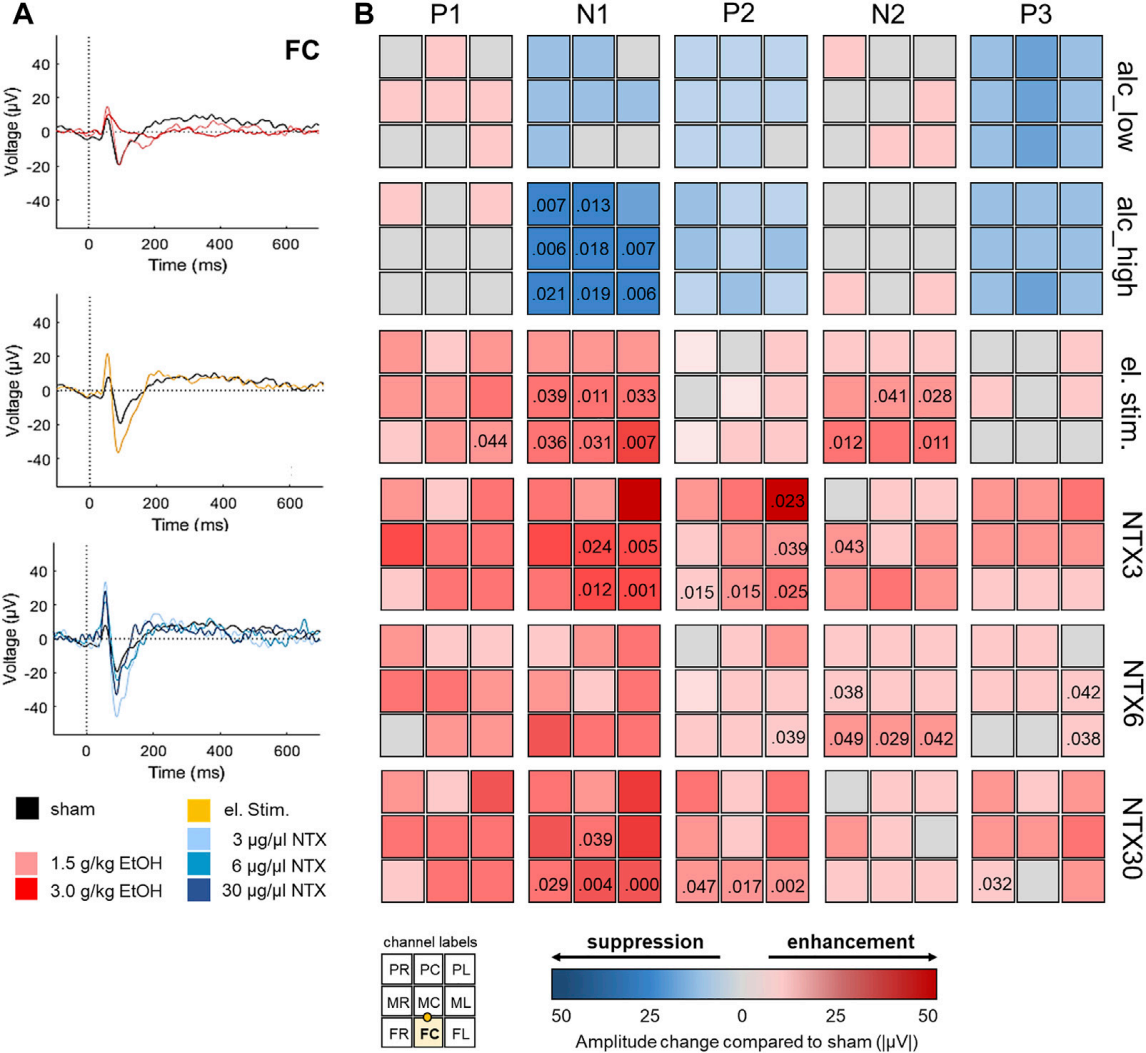
Impact of alcohol and implant-driven electrical and pharmacological brain stimulation on prefrontal neural activity. **(A)** Representative grand average deviant-minus-standard ERP difference curves at channel FC following administration of alcohol at 1.5 g/kg (n = 6, rose) and 3 g/kg (n = 9, red), electrical brain stimulation (n = 8, yellow), cortical delivery of naltrexone (NTX) at 3 μg/μl (n = 5, light blue), 6 μg/μl (n = 4, mid-blue), and 30 μg/μl (n = 5, dark blue) and untreated animals (sham, n = 10, black). **(B)** ERP amplitude differences between interventions and untreated animals at all channels. Implant channels are displayed in 3 × 3 matrices for each treatment (rows) and ERP component (columns) with suppressing or enhancing treatment effects on ERP amplitudes illustrated in blueish or reddish colors. The bottom left inset illustrates channel locations with the highlighted FC stimulation electrode and the microfluidic channel between electrodes FC and MC. Numbers indicate significant *p*-values (uncorrected).

Animals received a low (1.5 g/kg, n = 6) or a high (3.0 g/kg, n = 9) dose of EtOH. Behaviourally, the low dose induced just a slightly tottering gait, while the high dose resulted in ataxia and immobility. Paired *t*-tests revealed a delay of the N2 component at four channels in the low EtOH condition, but no significant impact on ERP amplitudes (Figure 4A,B; Supplementary Figure S4; Supplementary Table S4, S5). In contrast, high dosed EtOH significantly impaired neural functioning as reflected by a diminished N1 component Figure 4A,B; Supplementary Figure S4; Supplementary Table S7). ERP latencies for high EtOH were only analysed for the P1 component (Supplementary Table S6), as other ERP components were suppressed entirely.

Next, we investigated the effects of direct cortical stimulation on neural activity by applying biphasic, charge-imbalanced pulses. Such waveforms have been shown to provide a good compromise between effective neural activation and adverse effects such as tissue damage and dissolution of platinum electrodes (Merrill et al., 2005). Animals (n = 8) did not display behavioral changes upon stimulation, however, increased P1, N1 and N2 amplitudes at six channels indicated an enhancing effect of electrical stimulation on brain activity (Figure 4A,B; Supplementary Figure S5; Supplementary Table S9). For the N1 component, this effect was more pronounced in closer proximity to the stimulation site as revealed by paired *t*-tests using mean N1 responses combined for frontal (*t*(6) = −4.703, *p* = 0.003, |*d*| = 0.936) and mid-row electrodes (*t*(6) = −3.139, *p* = 0.020, |*d*| = 0.516) compared to posterior electrode sites (Figure 4B). ERP latencies were unchanged (Supplementary Table S8).

Finally, we investigated the effects of epicortical administration of NTX, an opioid receptor antagonist well established in recuperation but with just moderate effects in conventional oral application (Hendershot et al., 2017). We tested three different doses (3 µg (NTX3, n = 5), 6 µg (NTX6, n = 4), 30 µg (NTX30, n = 5)). Differences in animal behaviour upon NTX administration at either of the applied doses were not observed. NTX at 3 μg/μl and 30 μg/μl decreased latencies of P1 and N1 components (Supplementary Table S10, S14) and all concentrations displayed enhancing effects on amplitudes of N1, P2, N2 or P3 components (Figure 4A,B; Supplementary Figure S6; Supplementary Table S11, S13, S15). However, results remained significant following FDR-correction only at channels near the microchannel outlet for N1 amplitudes at a dose of 3 μg/μl (channels FC, MC, FL, ML) and 30 μg/μl (channels FC, FL) and for the P2 at channel FL and 30 μg/μl.

### Successful Single-Trial ERP Classification

Computing ERP grand averages in standard offline ERP analysis procedures is unsuitable for a neuroprosthetic device designed to operate in individuals in real-time. Thus, we applied machine learning algorithms to perform single-trial classification of ERP responses. In addition, we aimed to provide an accurate differentiation, of which treatment had been applied to each session for each individual.

**FIGURE 5:**
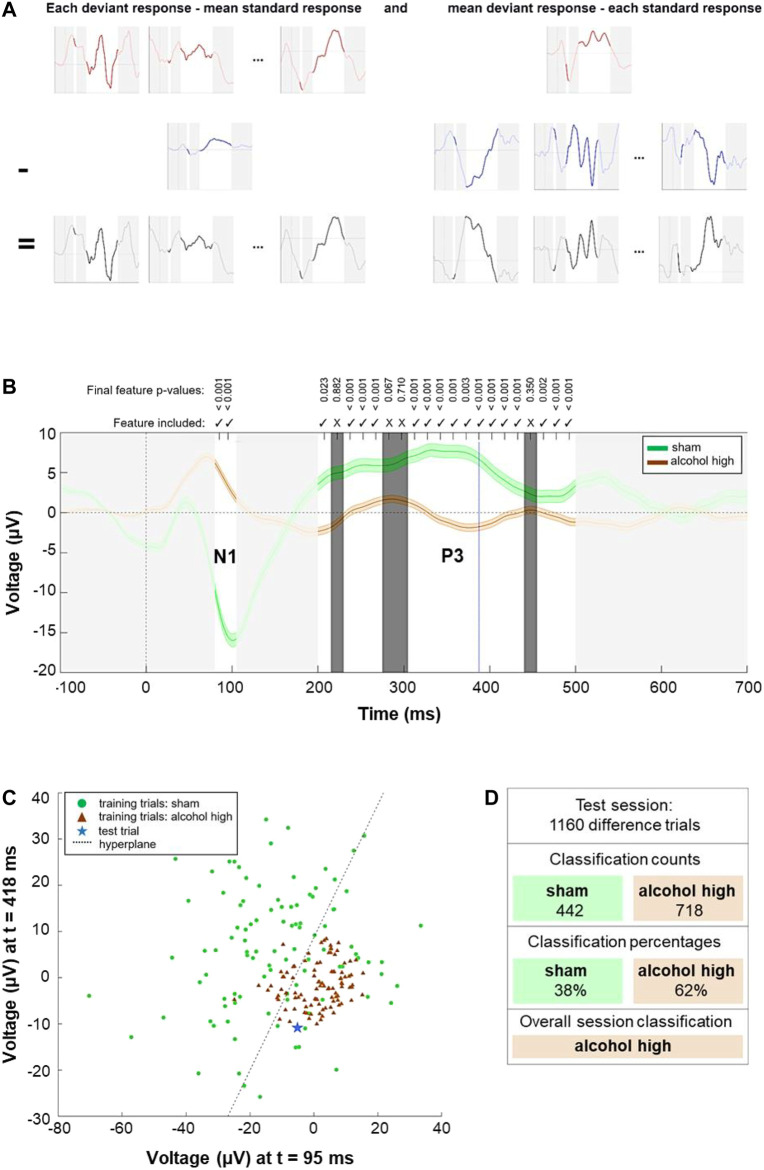
Approach to distinguish the treatment applied for each session. **(A)** Generation of training and test trials. Upper row (red): deviant ERP responses for a single animal and treatment, including the mean **(right)**. Mid row: standard ERP responses, including the mean **(left)**. Bottom row: resulting difference trials. Time ranges not used for classification are obscured in light grey. **(B)** Session-level feature selection results. Representing a example model for testing whether the animal’s session can be correctly classified as ‘alcohol high’ against ‘sham’ treatment. Solid green line: mean of all difference trials under ‘sham’ treatment. Solid brown line: mean of all difference trials under ‘alcohol high’ treatment. Shaded areas above and below these lines represent 1 standard error. Each individual feature represents the voltage at a given time point of each trial. For example, blue dotted line represents voltages of each trial at 388 ms post-stimulus. Time ranges, not eligible for classification, are obscured in light grey. Non-selected features are obscured in dark grey and marked with crosses above. Checkmarks for selected features and *p*-values of the last iteration are shown above. **(C)** Classification of single trials for which a simplified example of the linear discriminant classifier is employed. For visualization, the two features with the smallest *p*-values in the feature selection example shown in part **(B)** are extracted and the 100 samples closest to the arithmetic mean of each class are displayed. Green circles: samples from ‘sham’ treatment. Brown triangle: samples from ‘alcohol high’ treatment. The hyperplane generated by the classification model best separating the two classes is indicated as a dashed black line. An example test sample is shown as a blue star. This sample falls on the lower right side of the hyperplane, and it is correctly classified as an ‘alcohol high’ sample. **(D)** Overall session-level classification by the model. After each test trial (as generated in part **(A)** and tested as in part **(C)** with the features selected in part **(B)**, the majority of trials in this example was classified as ‘alcohol high’. Therefore, the overall prediction for the session is ‘alcohol high'.

Grand average data showed notable differences between sham recordings and EtOH treatments, especially in the N1 and P3 components. As these ERPs are known to peak at frontocentral electrode sites (Wronka et al., 2008; Campanella et al., 2014), FC data of these components were chosen for feature extraction based on voltage differences between sham recordings and treatments. The classification procedure providing the most accurate treatment differentiation is visualized in Figure 5. Table 1 comprises the accuracies of the resulting predictions for each one-vs-one session comparison. When comparing sham condition against treatment of 3 g/kg of EtOH, the correct treatment was predicted for 94.4% of sessions, with only one misclassification. Furthermore, brain states induced by electrical stimulation were correctly distinguished from all other conditions with a precision up to 100% (low EtOH dose). Pharmacological treatments with NTX were correctly classified in at least two-thirds of sessions for every comparison. Contingency tables reporting the predictions in each treatment comparison are shown in Supplementary Table S16.

**TABLE 1:** Percentage of sessions in which the treatment was accurately predicted for each one-vs-one treatment type comparison.

Treatment A	Treatment B	n	Session Classification Accuracy	*p*-value
Sham	alcohol high	9	94.4%	<0.001
Sham	alcohol low	6	66.7%	0.248
Alcohol low	alcohol high	6	83.3%	0.014
Sham	el. stimulation	8	75.0%	0.046
Alcohol high	el. stimulation	7	92.9%	0.001
Alcohol low	el. stimulation	6	100.0%	<0.001
Sham	NTX3	4	87.5%	0.029
Sham	NTX6	4	75.0%	0.103
Sham	NTX30	5	90.0%	0.001
Alcohol high	NTX3	3	100.0%	0.014
Alcohol high	NTX6	3	83.3%	0.083
Alcohol high	NTX30	4	75.0%	0.103
Alcohol low	NTX3	3	66.7%	0.410
Alcohol low	NTX6	3	66.7%	0.410
Alcohol low	NTX30	4	75.0%	0.103

### Biocompatibility of Implant Materials and Applied Treatments

**FIGURE 6:**
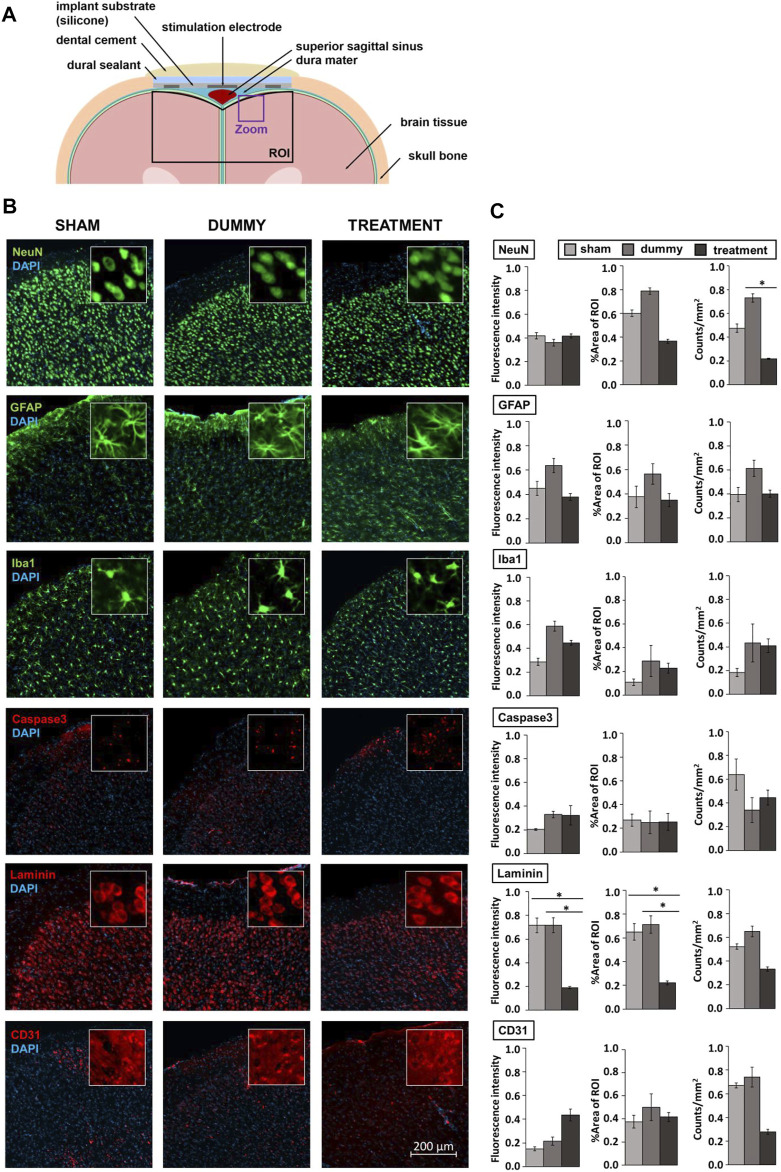
Immunohistochemical evaluation of implant and treatment tissue response. **(A)** Cross-section of rat brain and the biomedical implant at 3.2 mm anterior to bregma including the region of interest (ROI) defined for biocompatibility analysis following device explantation and the magnified view presented in **(B)**. **(B)** Representative microscopic images of the left motor cortex at 3.2 mm anterior to bregma next to the stimulation electrode. Insets are 50 × 50 µm squares. **(C)** Selected biotolerance indicators for each, six brain slices per staining of sham-operated animals (n = 3), rats that received an implant dummy (n = 3) and treatments (n = 7) are normalized and presented as mean ± SEM. Asterisks indicate significant differences (*p* < 0.05).

Finally, we investigated the biointegration of the device itself and the effects of the combined interventions on brain tissue after 4 weeks of implantation. This involved immunohistochemical evaluation of neuroinflammation by applying antibodies against GFAP and Iba1 and stainings of laminins and CD31, revealing cerebral vascular integrity. We furthermore investigated neuronal cell survival by using antibodies against the neuronal marker NeuN and caspase3, a mediator of programmed cell death. Immunostainings were performed on sham-operated animals without implants, animals that received a non-functional dummy implant and rats with an active implant that underwent EtOH injections, electrical stimulation and NTX delivery. Zoomed-in microscopic images of Figure 6 depict the immunoreactivity within the left motor cortex at 3.2 mm anterior to bregma next to the stimulation electrode (Figures 6A,B).

In line with other studies using ECoG implants (Schendel et al., 2014; Degenhart et al., 2016), we noticed fibrous tissue surrounding the electrode array leading to a slight depression of brain tissue underneath. In addition, we observed a lower number of NeuN positive cells in the treatment group (*F*(2,10) = 4.474, *p* = 0.045) indicative of an effect of treatment rather than caused by the device as animals that received an implant dummy did not display a significant difference in %area stained and numbers of NeuN positive cells compared to sham-operated rats (Figures 6B,C; Supplementary Table S17). Furthermore, no differences between groups were found for GFAP, Iba1 and caspase3, indicating that neither the dummy implant nor the treatments induced significantly enhanced inflammation or acute cell loss (Figures 6B,C; Supplementary Table S17). However, we observed treatment-related cellular alterations within the cerebral vascular system indicated by lower laminin expression in animals of the treatment group compared to sham-operated rats (fluorescence intensity: *F*(2,10) = 8.793, *p* = 0.021, %area of ROI: *F*(2,10) = 7.853, *p* = 0.042) and rats with implant dummies (fluorescence intensity: *F*(2,10) = 8.793, *p* = 0.021, %area of ROI: *F*(2,10) = 7.853, *p* = 0.020) Figures 6B,C; Supplementary Table S17)).

## Discussion

We established a multimodal neural interface combined with machine learning to acquire, modulate and classify ERP components in awake rats. By multimodal we mean, that our neural interface provides bidirectional functionality through 1) epicortical recording and 2) modulation of neural activity through implant-driven stimulation. This stimulation can be either electrical or pharmacological. This capacity of our device, to record neural signals together with the ability to stimulate in the electrical and chemical domains, contrasts conventional EEG systems, which provide recording only, and available stimulation techniques such as transcranial direct current stimulation or deep brain stimulation which are only able to stimulate. In addition, while available ECoG-based systems typically focus on sensorimotor brain areas, this study proposes the first-time application of an epicortical implant to target cognitive ERPs from higher-order prefrontal networks. The applied tools have potential applications in diagnosing and treating neuropsychiatric diseases and aim to pave the way for intelligent closed-loop neuroprostheses.

Custom electrode arrays were manufactured using a 3D printing technology with robot-controlled deposition of soft and conductive materials that allowed tailoring the implant layout to match the surface area above the mPFC. The flexible Pt-silicone electrodes of our arrays provided low impedances *in vitro*. Previous studies demonstrate that electrode impedances increase during the first weeks after implantation but decline over extended periods (Stiller et al., 2020). Similarly, we observed increasing impedances at 1 kHz of recording electrodes during the 3-weeks study period though we did not perform measurements over longer implantation durations to clarify if a decrease in impedances would also occur in our devices. Impedances of stimulation electrodes were lower than recording electrodes due to their larger surface area and remained stable over the entire study period. A potential explanation for this may be that electrical stimulation supposedly has a “rejuvenating” effect on the electrode-tissue interface by decreasing the tissue interface resistance resulting in improved signal quality and reduced electrode impedances (Johnson et al., 2005). The stability enabled reliable and high-quality field potential recordings.

Neuropsychiatric disorders are associated with disturbances in prefrontal brain activity that also translate into altered ERPs. Their monitoring is therefore increasingly supported to become part of the clinical routine (Campanella, 2013). We recorded ERPs appearing in the brain as early stages of auditory perception immediately after the onset of sounds. In healthy subjects, they differ depending on sound characteristics (e.g., pitch, loudness). Our bioelectronic implant was capable to reliably measure these ERP differences between rare deviant and frequent standard sounds in accordance with previous studies using similar paradigms (Sambeth et al., 2003, 2004; Ehlers et al., 2014). Likewise, we could successfully detect dose-dependent ERP changes following acute administration of alcohol. In line with available data from humans and rodents, the application of EtOH substantially affected the N1 component indicating impaired sensory gating and perceptual disturbances (Jääskeläinen et al., 1998; Gooding et al., 2013; Ehlers et al., 2014).

Although ERPs have been recognised as disease biomarkers (Campanella and Colin, 2014), their targeted monitoring and modulation is not the focus of current therapeutic interventions. In real-life, ERP abnormalities in the context of alcohol use disorders would express in reaction, e.g., to hearing the sound of opening a beer bottle or seeing people drinking alcohol. In an addicted individual, such alcohol-associated stimuli attract attention and challenge inhibitory abilities to withstand alcohol consumption (Campanella, 2016). These processes also express as altered neural activity patterns that are supposed to normalize when neuromodulation is applied to improve behavior control and decrease relapse risk. First studies using tDCS to correct neural disturbances in substance use disorders could positively affect N2 and P3 components (for review see (Habelt et al., 2020)). In our study, implant-driven direct cortical stimulation successfully enhanced the N1, P2 or N2 components at most channels, indicating improved auditory processing, sensory gating and enhanced perception of the deviant stimulus. Especially for the N1 component, this effect was more pronounced at electrodes closer to the stimulation site. Nguyen and Lin (Nguyen and Lin, 2014) further demonstrated that the frontal N1 might not only reflects passive sensory processing but also indicates motivational salience shown by increased N1 amplitudes in rats correctly responding to a reward-coupled deviant sound compared to miss responses. Therefore, modulating the N1 component could influence early inhibition of stimulus-activated actions (Debruille et al., 2019), which is relevant to addictive diseases because relapse, as previously mentioned, is triggered by drug cue-induced craving and impaired inhibitory control (Campanella, 2016). Abnormalities in stimulus-locked P2 and N2 have also been associated with drug users who discontinued treatment (Steele et al., 2014; Fink et al., 2016), suggesting that monitoring and modulation of these components could predict and influence treatment outcomes. The P3 component was not significantly influenced by stimulation here. However, the P3 is more pronounced in practical tasks involving active action planning and behaviour control than under passive conditions as applied here (Wronka et al., 2008).

Upon local delivery of NTX directly onto the PFC, we observed decreased latencies of P1 and N1 components (3 μg/μl and 30 μg/μl) and increased amplitudes of N1, P2, N2 or P3 (all doses applied) at several channels pointing to improved auditory processing. Although already approved in 1984 for addiction treatment (United.States Department of Health and Human Services, 2009), investigations of NTX effects on electrophysiological parameters are sparse and inconclusive. Earlier EEG studies could not detect an influence of systemic NTX application on the ERPs of our interest: in an auditory oddball paradigm with social drinkers, previous oral intake of NTX significantly reduced the late negative difference at 200–500 ms post-stimulus indicative for an NTX-induced impaired selective attention (Jääskeläinen et al., 1998). In contrast, a somewhat improved neural functioning by NTX was concluded from an increased language-related N4 in a semantic memory task performed by opioid addicts, long-term treated with NTX via an abdominal subcutaneous implant (Sheng-xi et al., 2009).

Towards autonomous identification of brain states evoked by neuromodulation and pharmacological treatment, single-trial ERP data underwent a machine learning procedure involving SWLDA. SWLDA has a strong track record in the classification of P3-related data and has again performed well here. The comparison between sham condition and a high dose of alcohol was classified correctly in 17 out of 18 sessions, with a high degree of statistical significance (*p* < 0.001). Interestingly, the administration of the two different doses of alcohol was accurately distinguished, supporting recent findings in which machine learning approaches revealed that ERPs correlate with individual differences in alcohol consumption behaviour in patients suffering from alcohol use disorders (O’Halloran et al., 2020). Machine learning techniques using ERP components have proven to differentiate patients from healthy controls also in schizophrenia (Neuhaus et al., 2013), attention deficit hyperactive disorder (Mueller et al., 2010) and autism (Eldridge et al., 2014). Importantly, our SWLDA approach could identify animals that received direct cortical stimulation. This is crucial for the development of closed-loop neuroprosthetics, which intend to reduce side effects and increase efficiency by switching on brain stimulation only if and as long as an abnormal neural activity is detected, adaptively adjusting stimulation parameters and switching off as soon as brain activity has been normalised (Parastarfeizabadi and Kouzani, 2017). Besides the NeuroPace® RNS® System for epilepsy, a closed-loop system utilizing a machine learning approach was successfully implemented for DBS in Parkinson’s disease to extract patient-specific cortical signals that indicate tremor-evoking movement and adjust stimulation voltage in real-time (Houston et al., 2019). ERPs and machine learning can thus support the diagnosis of psychiatric symptoms and contribute to developing predictions about disease progression and treatment outcomes in individual subjects, enabling a personalized and optimized therapy (Campanella, 2013; Thieme et al., 2020).

Although we have successfully demonstrated the suitability of our biomedical implant to obtain and modulate prefrontal ERPs, the safety of neural implants is crucial. Immunohistochemical analysis revealed good biocompatibility of the used implant materials, while animals in the treatment group displayed some variability in their cortical morphology. Note that animals received alcohol, electrical stimulation and local delivery of NTX. Therefore we cannot tell if the effects shown are caused by a specific treatment or result from interactions of treatments. However, this might reflect a clinical scenario where patients are treated with a combination of different approaches.

Differences in immunoreactivity were predominantly observed for laminins, a family of glycoproteins that belong to the extracellular matrix and are relevant to blood-brain-barrier and neuronal functioning (Lasek, 2016). Therefore, the detected decrease in expression of laminins likely affected neurons as well. Surgical interventions to implant biomedical interfaces include neural and vascular injury and typical foreign body reactions to the implanted devices (Prodanov and Delbeke, 2016). Here, the necessary incision of the dura mater to allow the influx of NTX solution likely induced some vascular damage causing the observed changes in laminin expression in some animals. However, vascular dysfunction and neuronal loss have also been associated with oxidative stress caused by alcohol abuse (Alikunju et al., 2011). Knabbe and colleagues (Knabbe et al., 2020) demonstrated that even a single intoxicating exposure to alcohol has long-lasting molecular and cellular effects on the brain. Electrical stimulation is capable of increasing the permeability of the blood-brain barrier as well. However, this effect is transient and reversible (Shin et al., 2020). Studies applying DBS suggest that electrical stimulation might regulate and even reduce neuroinflammation and apoptosis, thus exerting a neuroprotective effect (McKinnon et al., 2019). Further, alcohol-induced neurodegeneration has been associated with enhanced microglial and astrocytic expression (Saito et al., 2016). However, we did not observe significantly enhanced GFAP and Iba1 immunoreactivity in the treatment group. Since NTX has been shown to have anti-inflammatory and neuroprotective functions counteracting drug-induced activation of astrocytes, microglia and caspase3 (Hutchinson et al., 2011; Qin and Crews, 2012), we conjecture that NTX might have inhibited the potential damaging effects of repeated alcohol administration.

## Limitations

With the here applied passive auditory oddball paradigm, it is impossible to clarify if targeted ERP normalisation through an epicortical implant can re-establish lost behavior control. This would necessitate an experimental approach requiring subjects to actively respond to or inhibit their response to certain stimuli (Kutas et al., 2012). When applied e.g., in addiction models, stimuli can be paired with the availability and self-administration of drugs. The implant would then monitor and modulate neurophysiological correlates of drug-cue reactivity and associated drug seeking and consumption behaviour. As validated animal models are available (Nestler and Hyman, 2010), the experimental set-up presented here can be used straightway to target a wide range of neuropsychiatric disorders where disturbed prefrontal processing is a characteristic feature closely related to the clinical symptomatology, cognitive impairments and poor functional outcomes (Ramsay et al., 2020).

Neural measurements were currently performed following stimulation, and their analysis was carried out offline. These elements need to be combined to enable autonomous measurement, analysis, and modulation of neural activity in real-time. In addition, integration of a controllable liquid infusion unit might allow a more precise cortical drug delivery.

The used machine learning procedures to identify different treatment conditions were chosen on the assumption that linear classifiers perform well in the classification of brain signals compared to more complex approaches such as deep neural networks as they offer the possibility of overfitting, due to relatively small data sets usually being available (Lotte et al., 2007). However, in this scenario, the training data set is made up of trials from numerous animals. If a large cohort of animals is used, there may be enough training data to make deep learning an attractive alternative approach. In the current data set, the stimulation electrode was the only channel consistently recorded in all sessions for all animals. Increased reliability of channel recordings may be advantageous to classification, allowing more spatial information to be utilized. The current approach relies on patterns being learned in a group of animals and transferred to a different test animal. However, brain signals are known to suffer from variability (Polich, 2007). Further sessions of all conditions would allow calibration trials from the test animal’s other sessions to build a better model to generalize to a new session. With data from multiple sessions of each condition, it may be possible to build fully subject-dependent models for each animal, which may aid accuracy.

Finally, for analysis of treatment-induced tissue reactions, rats received a combination of interventions that hinder the allocation of the outcomes to a specific treatment. Moreover, biocompatibility evaluations should be performed after prolonged, clinically relevant implantation durations assessing persistent effects (Prodanov and Delbeke, 2016).

## Conclusion

Here, we established a 3D printed flexible and biocompatible epicortical implant for monitoring ERPs of the rat mPFC. Thereby, the manifestation of typical auditory ERPs expected to occur in healthy individuals as well as alcohol-induced neural impairments were successfully acquired through the device. Furthermore, implant-driven electrical and pharmacological stimulation enabled a measurable modulation of neural activity that has been classified autonomously through new machine learning algorithms able to deal with sparse data. Though further investigations are needed to demonstrate the utility of our approach for functional recovery from pathological behaviour, these results are promising and underline the great potential of bioelectronic medicine in the diagnosis and therapy of mental disorders.

## Data Availability

The raw data supporting the conclusions of this article will be made available by the authors, without undue reservation.
