# Supplementary material for: A Multimodal Neuroprosthetic Interface to Record, Modulate and Classify Electrophysiological Biomarkers Relevant to Neuropsychiatric Disorders
Source: Front Bioeng Biotechnol. 2021 Nov 3;9:770274. doi: 10.3389/fbioe.2021.770274 (PMC8595111; doi:10.3389/fbioe.2021.770274)
Supplement: Supplementary file 1 [file DataSheet1.PDF]

Supplementary Material

This PDF file includes:

Figures S1 to S6
Tables S1 to S17

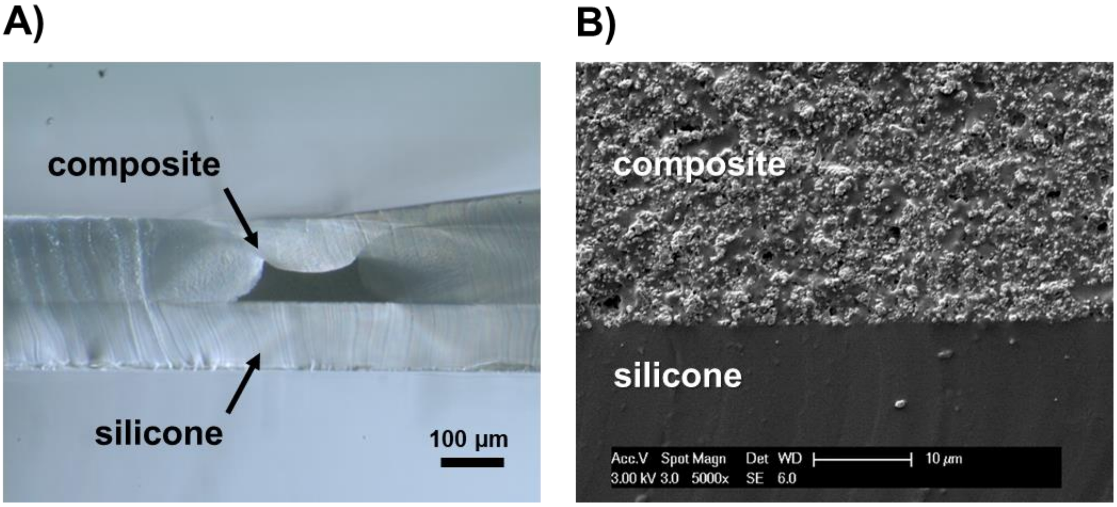

Figure S1 Implant cross sections
A) An optical micrograph of a cross section of a printed array showing an embedded interconnect. Although the layers used for the construction of the array are visible, no delamination or gap defects are observable. The silicone layers form a good bond before thermally induced polymerization. **B)** Scanning electron micrograph showing detail of the interface between a silicone insulator layer and the conductive composite.

Table S1 Antibodies used to investigate implant and treatment-specific tissue reactions

Primary antibody	conc.	Abcam-ID	Secondary antibody + fluorescent dye	conc.	Abcam-ID
goat anti-GFAP	1:500	ab53554	donkey anti-goat Alexa Fluor 488	1:500	ab150133
rabbit anti-Laminin	1:500	ab7463	donkey anti-rabbit Alexa Fluor 647		ab150067
goat anti-Iba1	1:200	ab5076	donkey anti-goat Alexa Fluor 488	1:500	ab150133
rabbit anti-Caspase3	1:500	ab13847	donkey anti-rabbit Alexa Fluor 647		ab150067
rabbit anti-NeuN	1:500	ab177487	goat anti-rabbit Alexa Fluor 488	1:500	ab150081
mouse anti-CD31	1:200	ab64543	goat anti-mouse Alexa Fluor 680		ab186694

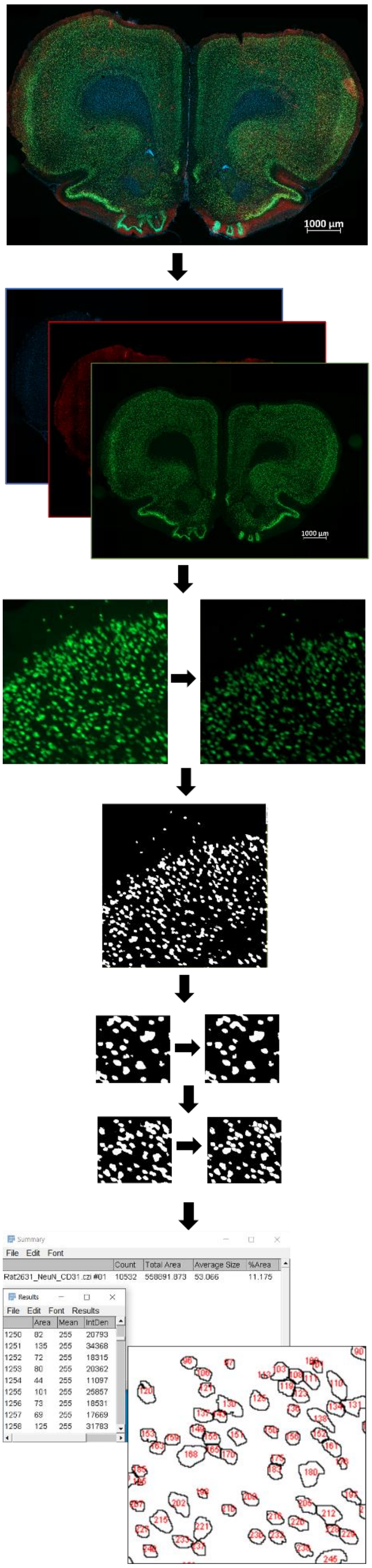

Original image

1. Split channels

2. Subtract background

3. Apply global threshold → binary image

4. Improve object quality
(e.g. by using Fiji´s „open“ command to remove very small objects)

5. Split clumped objects by using the „watershed“ command

6. Count objects and measure object properties (e.g. intensity)

Figure S2 Image processing steps and analysis of immunofluorescence tissue staining

Table S2 Impedances at 1 kHz of recording and stimulation electrodes *in vitro* and *in vivo*

	No. of implants tested	% functional electrodes	Impedance at 1 kHz (kΩ) Mean ± SEM	
			Recording El.	Stimulation El.
in vitro	10	100	10.14 ± 1.96	4.36 ± 1.41
in vivo, day 3	10	93	65.10 ± 7.08	41.71 ± 11.91
in vivo, day 6	10	93	64.04 ± 7.16	53.97 ± 13.22
in vivo, day 9	8	92	96.05 ± 12.38	45.96 ± 10.11
in vivo, day 12	7	81	122.40 ± 10.62	53.02 ± 14.13
in vivo, day 15	5	87	120.39 ± 18.72	32.54 ± 4.15
in vivo, day 18	5	82	161.29 ± 23.20	33.90 ± 5.50
in vivo, day 21	5	80	188.79 ± 33.41	43.63 ± 11.90

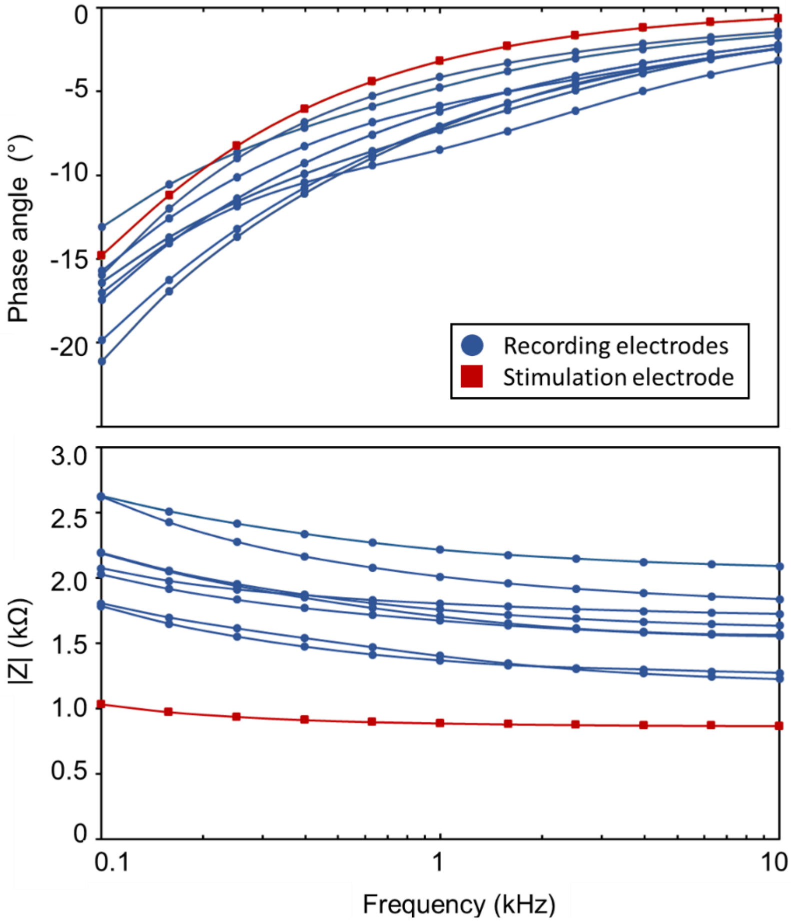

Figure S3 Impedance spectroscopy of soft platinum electrodes. Modulus and phase angle of electrode impedances recorded in PBS of one representative neural implant consisting of n = 8 recording electrodes (blue) and one stimulation electrode (red).

Table S3 One-sample t-test of grand average ERP amplitudes of the difference curve (deviant-minus-standard) against zero value representing the different neural activity in response to the two sounds.

Electrode	ERP component	Amplitude mean ± SEM (µV)			df	*t*	*p*	FDR-*p*	|*d*|
PR	P1	6.718	±	2.499	8	2.688	***0.028***	***0.042***	0.896
	N1	-16.158	±	4.348		-3.716	***0.006***	***0.014***	1.239
	P2	12.294	±	4.959		2.479	***0.038***	0.068	0.826
	N2	-6.280	±	2.710		-2.317	***0.049***	0.088	0.772
	P3	10.992	±	2.759		3.984	***0.004***	***0.009***	1.328
MR	P1	9.585	±	3.260	9	2.940	***0.016***	***0.036***	0.930
	N1	-16.874	±	5.111		-3.301	***0.009***	***0.014***	1.044
	P2	14.559	±	5.533		2.632	***0.027***	0.068	0.832
	N2	-5.288	±	2.487		-2.126	0.062	0.093	0.672
	P3	12.467	±	3.759		3.317	***0.009***	***0.012***	1.049
FR	P1	8.988	±	2.002	8	4.488	***0.002***	***0.018***	1.496
	N1	-19.182	±	5.966		-3.215	***0.012***	***0.014***	1.072
	P2	12.939	±	6.378		2.029	0.077	0.077	0.676
	N2	-6.521	±	2.725		-2.393	***0.044***	0.088	0.798
	P3	13.930	±	4.603		3.027	***0.016***	***0.018***	1.009
FC	P1	7.771	±	3.322	9	2.339	***0.044***	0.050	0.740
	N1	-19.521	±	5.654		-3.452	***0.007***	***0.014***	1.092
	P2	12.755	±	5.834		2.186	0.057	0.073	0.691
	N2	-6.663	±	4.670		-1.427	0.187	0.187	0.451
	P3	16.675	±	4.462		3.737	***0.005***	***0.009***	1.182
PC	P1	9.169	±	2.496	7	3.674	***0.008***	***0.024***	1.299
	N1	-17.648	±	5.291		-3.335	***0.012***	***0.014***	1.179
	P2	12.106	±	4.245		2.852	***0.025***	0.068	1.008
	N2	-7.429	±	3.608		-2.059	0.078	0.100	0.728
	P3	15.055	±	1.953		7.710	***0.001***	***0.005***	2.726
MC	P1	5.453	±	2.409	8	2.264	0.053	0.053	0.755
	N1	-16.528	±	4.759		-3.473	***0.008***	***0.014***	1.158
	P2	12.911	±	5.379		2.400	***0.043***	0.068	0.800
	N2	-7.484	±	4.082		-1.833	0.104	0.117	0.611
	P3	13.887	±	3.796		3.659	***0.006***	***0.009***	1.220
FL	P1	7.724	±	2.103	9	3.673	***0.005***	***0.023***	1.162
	N1	-18.192	±	4.166		-4.367	***0.002***	***0.014***	1.381
	P2	11.388	±	4.912		2.318	***0.046***	0.068	0.733
	N2	-7.630	±	3.035		-2.514	***0.033***	0.088	0.795
	P3	14.140	±	3.220		4.392	***0.002***	***0.006***	1.389
ML	P1	7.016	±	2.543	8	2.759	***0.025***	***0.042***	0.920
	N1	-17.426	±	5.582		-3.122	***0.014***	***0.014***	1.041
	P2	12.228	±	5.734		2.132	0.066	0.074	0.711
	N2	-7.268	±	2.336		-3.111	***0.014***	0.088	1.037
	P3	11.393	±	3.837		2.969	***0.018***	***0.018***	0.990
PL	P1	5.608	±	2.187	8	2.564	***0.033***	***0.042***	0.855
	N1	-13.043	±	3.602		-3.621	***0.007***	***0.014***	1.207
	P2	8.651	±	2.475		3.495	***0.008***	0.068	1.165
	N2	-6.746	±	2.453		-2.750	***0.025***	0.088	0.917
	P3	10.077	±	2.132		4.726	***0.001***	***0.005***	1.575

SEM: standard error of the mean, df: degrees of freedom related to available channel data, FDR-*p*: false discovery rate-adjusted *p*-value, $|d|$: effect size Cohen´s *d* with $|d| \geq 0.2$ = small, $|d| \geq 0.5$ = medium and $|d| > 0.8$ = large effects. Significant *p*-values given in bold, italic.

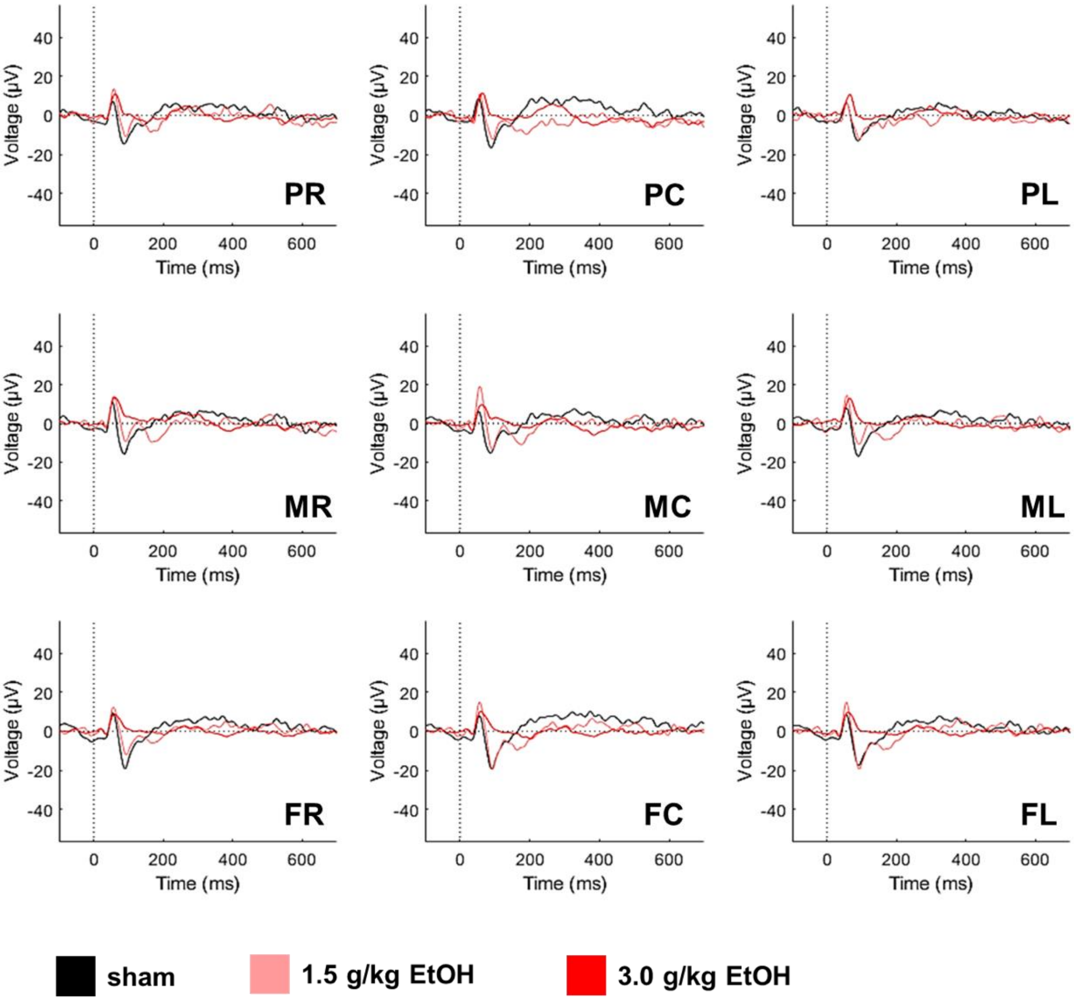

Figure S4 Grand average deviant-minus-standard ERP difference curves following administration of alcohol at 1.5 g/kg (n = 6, rose), 3 g/kg (n = 9, red) and untreated animals (sham, n = 10, black).

frontiers

Table S4 Statistical analysis of ERP latencies following alcohol administration at 1.5 mg/kg

Electrode	ERP component	Comparison	Latency mean	±	SEM (ms)	df	*t*	*p*	FDR-*p*	|*d*|
PR	P1	sham	57.534	±	2.873	4	0.102	0.924	0.963	0.046
		alc_low	57.200	±	0.501					
	N1	sham	94.600	±	4.026	4	0.757	0.491	0.785	0.339
		alc_low	90.668	±	2.273					
	P2	sham	123.668	±	1.032	4	0.730	0.506	0.980	0.327
		alc_low	121.200	±	2.837					
	N2	sham	142.466	±	2.062	4	-4.660	***0.010***	***0.043***	2.084
		alc_low	168.734	±	4.510					
	P3	sham	291.200	±	49.814	4	-0.403	0.707	0.901	0.180
		alc_low	321.066	±	52.007					
MR	P1	sham	58.268	±	4.443	4	0.181	0.865	0.963	0.081
		alc_low	57.400	±	0.520					
	N1	sham	95.998	±	4.017	4	1.126	0.323	0.785	0.503
		alc_low	90.000	±	1.719					
	P2	sham	118.932	±	2.971	4	-0.514	0.635	0.980	0.230
		alc_low	121.534	±	2.907					
	N2	sham	150.066	±	6.247	4	-1.851	0.138	0.196	0.828
		alc_low	167.400	±	3.817					
	P3	sham	367.466	±	33.286	4	-0.325	0.762	0.901	0.145
		alc_low	393.000	±	48.971					
FR	P1	sham	61.780	±	3.451	5	1.396	0.222	0.665	0.570
		alc_low	56.722	±	0.832					
	N1	sham	95.278	±	2.820	5	0.462	0.664	0.785	0.189
		alc_low	93.110	±	3.525					
	P2	sham	119.055	±	2.343	5	-0.447	0.673	0.980	0.183
		alc_low	120.833	±	2.461					
	N2	sham	138.167	±	4.886	5	-4.114	***0.009***	***0.043***	1.680
		alc_low	168.778	±	3.954					
	P3	sham	355.722	±	33.020	5	0.266	0.801	0.901	0.109
		alc_low	340.445	±	43.632					
FC	P1	sham	63.002	±	3.386	5	1.682	0.153	0.665	0.687
		alc_low	56.835	±	0.922					
	N1	sham	95.388	±	2.854	5	0.368	0.728	0.785	0.150
		alc_low	94.332	±	2.241					
	P2	sham	121.388	±	2.784	5	-0.171	0.871	0.909	0.070
		alc_low	122.112	±	2.452					
	N2	sham	137.555	±	6.373	5	-1.689	0.152	0.196	0.689
		alc_low	157.557	±	5.840					
	P3	sham	348.388	±	40.594	5	-0.537	0.614	0.901	0.219
		alc_low	390.777	±	40.015					
PC	P1	sham	57.750	±	2.968	3	0.054	0.960	0.963	0.027
		alc_low	57.585	±	0.533					
	N1	sham	92.833	±	4.494	3	-0.342	0.755	0.785	0.171
		alc_low	94.748	±	3.788					
	P2	sham	119.833	±	3.539	3	-0.027	0.980	0.980	0.014
		alc_low	120.000	±	3.391					
	N2	sham	144.585	±	2.537	3	-3.838	***0.031***	0.094	1.919
		alc_low	169.168	±	4.652					
	P3	sham	254.418	±	30.604	3	-1.420	0.251	0.901	0.710
		alc_low	341.918	±	55.677					

Table S4 Statistical analysis of ERP latencies following alcohol administration at 1.5 mg/kg (continued)

Electrode	ERP component	Comparison	Latency mean ± SEM (ms)			df	*t*	*p*	FDR-*p*	|*d*|
MC	P1	sham	57.668	±	5.359	4	0.050	0.963	0.963	0.025
		alc_low	57.418	±	0.761					
	N1	sham	88.418	±	4.043	4	-0.714	0.527	0.785	0.357
		alc_low	91.750	±	1.624					
	P2	sham	120.066	±	2.225	4	-0.156	0.883	0.980	0.070
		alc_low	120.734	±	2.585					
	N2	sham	145.915	±	8.909	4	-2.688	0.075	0.134	1.344
		alc_low	173.000	±	4.778					
	P3	sham	346.418	±	57.944	4	-0.279	0.799	0.901	0.139
		alc_low	372.250	±	50.862					
FL	P1	sham	62.608	±	3.086	5	1.927	0.112	0.665	0.787
		alc_low	56.165	±	0.811					
	N1	sham	95.500	±	3.241	5	0.288	0.785	0.785	0.117
		alc_low	94.498	±	2.456					
	P2	sham	121.500	±	2.500	5	0.056	0.957	0.980	0.023
		alc_low	121.278	±	2.318					
	N2	sham	142.445	±	6.018	5	-0.508	0.633	0.712	0.207
		alc_low	147.833	±	8.185					
	P3	sham	330.112	±	40.255	5	-0.650	0.544	0.901	0.265
		alc_low	377.110	±	32.708					
ML	P1	sham	60.866	±	3.932	4	0.972	0.386	0.868	0.435
		alc_low	57.134	±	0.441					
	N1	sham	93.466	±	3.543	4	-0.474	0.660	0.785	0.212
		alc_low	94.998	±	2.864					
	P2	sham	122.334	±	1.636	4	0.729	0.506	0.980	0.326
		alc_low	120.066	±	2.299					
	N2	sham	148.068	±	7.370	4	-2.833	***0.047***	0.106	1.267
		alc_low	165.732	±	4.942					
	P3	sham	354.468	±	41.759	4	-0.100	0.925	0.925	0.045
		alc_low	361.400	±	39.904					
PL	P1	sham	56.780	±	2.730	2	0.116	0.919	0.963	0.067
		alc_low	56.443	±	1.660					
	N1	sham	95.553	±	4.802	2	0.397	0.730	0.785	0.229
		alc_low	92.890	±	1.947					
	P2	sham	124.557	±	0.443	2	1.171	0.362	0.980	0.676
		alc_low	119.000	±	4.583					
	N2	sham	148.890	±	10.970	2	-0.075	0.947	0.947	0.044
		alc_low	150.443	±	10.229					
	P3	sham	289.110	±	19.065	2	-0.665	0.575	0.901	0.384
		alc_low	329.113	±	41.469					

SEM: standard error of the mean, df: degrees of freedom related to available channel data, FDR-*p*: false discovery rate-adjusted p-value, $|d|$: effect size Cohen´s *d* with $|d| \geq 0.2$ = small, $|d| \geq 0.5$ = medium and $|d| > 0.8$ = large effects. Significant *p*-values given in bold, italic

Table S5 Statistical analysis of ERP amplitudes following alcohol administration at 1.5 mg/kg

Electrode	ERP component	Comparison	Amplitude mean ± SEM (μV)			df	*t*	*p*	FDR-*p*	|*d*|
PR	P1	sham	9.840	±	3.118	4	-0.205	0.847	0.953	0.092
		alc_low	11.188	±	5.332					
	N1	sham	-19.430	±	7.045	4	-1.914	0.128	0.577	0.856
		alc_low	-10.088	±	4.278					
	P2	sham	17.552	±	8.386	4	1.373	0.242	0.613	0.614
		alc_low	10.104	±	3.420					
	N2	sham	-5.226	±	4.264	4	0.736	0.502	0.753	0.329
		alc_low	-9.474	±	2.374					
	P3	sham	14.722	±	4.049	4	0.748	0.496	0.547	0.334
		alc_low	9.874	±	4.562					
MR	P1	sham	7.990	±	2.093	4	-0.589	0.587	0.817	0.264
		alc_low	12.098	±	5.985					
	N1	sham	-17.078	±	9.716	4	-1.143	0.317	0.687	0.511
		alc_low	-8.606	±	5.161					
	P2	sham	17.612	±	10.779	4	1.183	0.302	0.613	0.529
		alc_low	8.688	±	3.465					
	N2	sham	-6.548	±	3.023	4	0.871	0.433	0.753	0.390
		alc_low	-10.320	±	2.663					
	P3	sham	14.708	±	7.071	4	0.658	0.547	0.547	0.294
		alc_low	8.418	±	3.784					
FR	P1	sham	8.433	±	2.551	5	-0.505	0.635	0.817	0.206
		alc_low	10.760	±	3.656					
	N1	sham	-19.247	±	8.844	5	-1.216	0.278	0.687	0.497
		alc_low	-11.735	±	3.989					
	P2	sham	15.102	±	9.707	5	0.929	0.396	0.613	0.379
		alc_low	8.703	±	3.239					
	N2	sham	-5.475	±	3.673	5	0.333	0.753	0.753	0.136
		alc_low	-7.060	±	2.751					
	P3	sham	17.905	±	6.333	5	1.172	0.294	0.441	0.479
		alc_low	8.633	±	2.744					
FC	P1	sham	9.598	±	4.741	5	-0.567	0.595	0.817	0.232
		alc_low	13.010	±	3.848					
	N1	sham	-18.505	±	9.305	5	-0.042	0.968	0.968	0.017
		alc_low	-18.115	±	5.138					
	P2	sham	16.120	±	9.587	5	0.538	0.613	0.613	0.220
		alc_low	11.673	±	3.579					
	N2	sham	-3.305	±	7.333	5	0.921	0.400	0.753	0.376
		alc_low	-10.635	±	2.297					
	P3	sham	22.497	±	6.439	5	1.840	0.125	0.284	0.751
		alc_low	9.138	±	2.461					
PC	P1	sham	10.908	±	3.926	3	-1.196	0.318	0.817	1.196
		alc_low	15.900	±	3.280					
	N1	sham	-18.288	±	8.247	3	-1.022	0.382	0.687	1.196
		alc_low	-10.923	±	6.139					
	P2	sham	13.795	±	8.376	3	0.760	0.503	0.613	0.380
		alc_low	8.793	±	1.850					
	N2	sham	-7.298	±	4.892	3	0.439	0.690	0.753	0.401
		alc_low	-10.370	±	3.698					
	P3	sham	17.025	±	3.494	3	2.289	0.106	0.284	2.289
		alc_low	5.725	±	3.348					

Table S5 Statistical analysis of ERP amplitudes following alcohol administration at 1.5 mg/kg (continued)

Electrode	ERP component	Comparison	Amplitude mean ± SEM (μV)			df	*t*	*p*	FDR-*p*	|*d*|
MC	P1	sham	6.863	±	3.339	4	-1.422	0.250	0.817	0.711
		alc_low	14.938	±	2.728					
	N1	sham	-16.268	±	9.526	4	-0.526	0.636	0.817	0.263
		alc_low	-11.733	±	4.078					
	P2	sham	14.868	±	9.747	4	0.837	0.450	0.613	0.374
		alc_low	10.292	±	4.317					
	N2	sham	-5.895	±	8.137	4	0.374	0.733	0.753	0.187
		alc_low	-9.860	±	3.434					
	P3	sham	21.170	±	6.701	4	2.103	0.126	0.284	1.052
		alc_low	3.148	±	2.724					
FL	P1	sham	8.388	±	2.577	5	-0.908	0.406	0.817	-0.371
		alc_low	12.713	±	3.660					
	N1	sham	-16.088	±	5.972	5	0.344	0.745	0.838	0.141
		alc_low	-18.363	±	5.413					
	P2	sham	14.172	±	8.120	5	0.547	0.608	0.613	0.223
		alc_low	11.352	±	4.052					
	N2	sham	-3.997	±	3.946	5	1.671	0.156	0.753	0.682
		alc_low	-12.325	±	1.896					
	P3	sham	18.345	±	4.550	5	1.445	0.208	0.375	0.590
		alc_low	8.667	±	3.242					
ML	P1	sham	8.068	±	2.225	4	-0.709	0.518	0.817	0.317
		alc_low	12.088	±	4.980					
	N1	sham	-14.718	±	6.741	4	-1.933	0.125	0.577	0.864
		alc_low	-10.380	±	4.961					
	P2	sham	14.056	±	3.941	4	0.800	0.468	0.613	0.358
		alc_low	9.772	±	2.101					
	N2	sham	-4.258	±	2.923	4	1.187	0.301	0.753	0.531
		alc_low	-9.966	±	2.787					
	P3	sham	14.440	±	6.846	4	0.894	0.422	0.543	0.400
		alc_low	6.224	±	3.674					
PL	P1	sham	4.903	±	1.231	2	0.035	0.976	0.976	0.020
		alc_low	4.677	±	5.671					
	N1	sham	-12.883	±	6.233	2	-0.582	0.619	0.817	0.336
		alc_low	-11.457	±	5.466					
	P2	sham	5.617	±	0.846	2	-0.725	0.544	0.613	0.419
		alc_low	6.873	±	0.933					
	N2	sham	-8.583	±	4.394	2	-0.536	0.646	0.753	0.309
		alc_low	-7.400	±	2.212					
	P3	sham	10.260	±	1.745	2	2.568	0.124	0.284	1.483
		alc_low	5.137	±	3.300					

SEM: standard error of the mean, df: degrees of freedom related to available channel data, FDR-*p*: false discovery rate-adjusted p-value, |*d*|: effect size Cohen´s *d* with $|d| \geq 0.2$ = small, $|d| \geq 0.5$ = medium and $|d| > 0.8$ = large effects. Significant *p*-values given in bold, italic

Table S6 Statistical analysis of P1 latencies following alcohol administration at 3 mg/kg

Electrode	ERP component	Comparison	Latency mean ± SEM (ms)			df	*t*	*p*	FDR-*p*	|*d*|
PR	P1	sham	56.381	±	2.118	6	-0.248	0.812	0.883	0.094
		alc_high	57.809	±	5.428					
MR	P1	sham	56.293	±	2.829	7	-2.114	0.072	0.428	0.747
		alc_high	61.835	±	2.981					
FR	P1	sham	59.334	±	2.556	8	0.503	0.629	0.883	0.168
		alc_high	56.370	±	4.283					
FC	P1	sham	60.224	±	2.606	8	0.542	0.603	0.883	0.181
		alc_high	57.074	±	4.196					
PC	P1	sham	56.381	±	2.506	6	0.240	0.819	0.883	0.091
		alc_high	54.810	±	6.812					
MR	P1	sham	55.793	±	2.744	7	0.570	0.587	0.883	0.201
		alc_high	51.999	±	5.139					
FL	P1	sham	59.790	±	2.706	7	0.492	0.638	0.883	0.174
		alc_high	56.541	±	4.731					
ML	P1	sham	58.331	±	2.079	6	-1.981	0.095	0.428	0.749
		alc_high	63.619	±	2.490					
PL	P1	sham	57.002	±	1.835	4	-0.156	0.883	0.883	0.070
		alc_high	58.334	±	7.496					

SEM: standard error of the mean, df: degrees of freedom related to available channel data, FDR-*p*: false discovery rate-adjusted p-value, |*d*|: effect size Cohen´s *d* with $|d| \geq 0.2$ = small, $|d| \geq 0.5$ = medium and $|d| > 0.8$ = large effects. Significant *p*-values given in bold, italic

Table S7 Statistical analysis of ERP amplitudes following alcohol administration at 3 mg/kg

Electrode	ERP component	Comparison	Amplitude mean ± SEM (μV)			df	*t*	*p*	FDR-*p*	|*d*|
PR	P1	sham	9.491	±	2.171	6	-1.621	0.156	0.737	0.613
		alc_high	13.659	±	4.004					
	N1	sham	-19.109	±	5.040	6	-4.057	***0.007***	***0.017***	1.534
		alc_high	-0.379	±	2.113					
	P2	sham	15.106	±	6.019	6	1.686	0.143	0.217	0.637
		alc_high	4.093	±	2.241					
	N2	sham	-6.009	±	3.140	6	-0.063	0.952	0.999	0.024
		alc_high	-5.694	±	2.620					
	P3	sham	11.904	±	3.336	6	0.747	0.483	0.483	0.282
		alc_high	9.370	±	2.331					
MR	P1	sham	12.434	±	3.356	7	-0.442	0.672	0.737	0.156
		alc_high	14.398	±	3.754					
	N1	sham	-19.768	±	6.000	7	-3.909	***0.006***	***0.017***	1.382
		alc_high	1.633	±	2.954					
	P2	sham	17.271	±	6.618	7	1.863	0.105	0.217	0.659
		alc_high	4.673	±	1.955					
	N2	sham	-4.268	±	2.859	7	-0.966	0.366	0.918	0.342
		alc_high	-1.264	±	2.537					
	P3	sham	14.219	±	4.496	7	0.921	0.388	0.460	0.326
		alc_high	9.676	±	2.089					
FR	P1	sham	8.988	±	2.002	8	-0.347	0.737	0.737	0.116
		alc_high	10.024	±	2.680					
	N1	sham	-19.182	±	5.966	8	-2.854	***0.021***	***0.024***	0.951
		alc_high	0.157	±	2.091					
	P2	sham	12.939	±	6.378	8	1.296	0.231	0.246	0.432
		alc_high	4.357	±	1.511					
	N2	sham	-6.521	±	2.725	8	-1.329	0.221	0.918	0.443
		alc_high	-2.151	±	1.446					
	P3	sham	13.930	±	4.603	8	1.722	0.123	0.223	0.574
		alc_high	5.709	±	1.255					
FC	P1	sham	9.572	±	3.120	8	-0.405	0.696	0.737	0.135
		alc_high	11.158	±	2.684					
	N1	sham	-20.266	±	6.267	8	-2.938	***0.019***	***0.024***	0.979
		alc_high	0.568	±	2.246					
	P2	sham	13.906	±	6.394	8	1.525	0.166	0.217	0.508
		alc_high	3.758	±	1.319					
	N2	sham	-5.622	±	5.090	8	-0.319	0.758	0.975	0.106
		alc_high	-3.806	±	1.705					
	P3	sham	17.789	±	4.831	8	2.266	0.053	0.223	0.755
		alc_high	5.312	±	1.462					
PC	P1	sham	10.797	±	2.184	6	-1.154	0.292	0.737	0.436
		alc_high	14.503	±	4.315					
	N1	sham	-19.616	±	5.671	6	-3.494	***0.013***	***0.023***	1.321
		alc_high	-0.211	±	2.334					
	P2	sham	13.599	±	4.589	6	1.927	0.102	0.217	0.728
		alc_high	4.277	±	1.554					
	N2	sham	-8.216	±	4.065	6	-0.537	0.611	0.975	0.203
		alc_high	-5.477	±	2.471					
	P3	sham	15.513	±	2.192	6	2.153	0.075	0.223	0.814
		alc_high	9.973	±	2.680					

Table S7 Statistical analysis of ERP amplitudes following alcohol administration at 3 mg/kg (continued)

Electrode	ERP component	Comparison	Amplitude mean ± SEM (µV)			t	*df*	*p*	FDR-*p*	|*d*|
MC	P1	sham	7.233	±	1.842	7	-0.862	0.417	0.737	0.305
		alc_high	10.925	±	3.796					
	N1	sham	-17.371	±	5.311	7	-3.091	***0.018***	***0.024***	1.093
		alc_high	-0.726	±	2.571					
	P2	sham	14.033	±	5.965	7	1.691	0.135	0.217	0.598
		alc_high	5.164	±	1.826					
	N2	sham	-6.455	±	4.479	7	-0.420	0.687	0.975	0.148
		alc_high	-4.061	±	2.114					
	P3	sham	14.969	±	4.125	7	1.749	0.124	0.223	0.619
		alc_high	6.629	±	1.924					
FL	P1	sham	8.606	±	2.029	7	-0.716	0.497	0.737	0.253
		alc_high	11.191	±	3.470					
	N1	sham	-20.750	±	4.759	7	-3.831	***0.006***	***0.017***	1.354
		alc_high	-0.541	±	2.206					
	P2	sham	12.595	±	6.094	7	1.534	0.169	0.217	0.542
		alc_high	4.483	±	1.644					
	N2	sham	-8.550	±	3.089	7	-1.069	0.320	0.918	0.378
		alc_high	-4.219	±	2.014					
	P3	sham	14.973	±	3.976	7	1.934	0.094	0.223	0.684
		alc_high	6.886	±	1.144					
ML	P1	sham	9.034	±	2.676	6	-0.989	0.361	0.737	0.374
		alc_high	12.729	±	3.260					
	N1	sham	-20.616	±	6.715	6	-3.970	***0.007***	***0.017***	1.500
		alc_high	0.640	±	1.571					
	P2	sham	14.637	±	7.201	6	1.863	0.112	0.217	0.704
		alc_high	3.357	±	1.325					
	N2	sham	-6.974	±	2.623	6	-0.889	0.408	0.918	0.336
		alc_high	-3.687	±	2.209					
	P3	sham	12.224	±	4.934	6	1.053	0.333	0.460	0.398
		alc_high	7.113	±	1.314					
PL	P1	sham	6.024	±	1.046	4	-1.335	0.253	0.737	0.597
		alc_high	11.606	±	4.361					
	N1	sham	-13.230	±	3.655	4	-1.646	0.175	0.175	0.736
		alc_high	-2.934	±	3.066					
	P2	sham	9.326	±	3.708	4	1.358	0.246	0.246	1.026
		alc_high	5.096	±	1.769					
	N2	sham	-4.938	±	3.371	4	0.001	0.999	0.999	0.001
		alc_high	-4.944	±	3.062					
	P3	sham	12.424	±	3.148	4	0.922	0.409	0.460	0.412
		alc_high	8.740	±	2.103					

SEM: standard error of the mean, df: degrees of freedom related to available channel data, FDR-*p*: false discovery rate-adjusted p-value, |*d*|: effect size Cohen´s *d* with $|d| \geq 0.2$ = small, $|d| \geq 0.5$ = medium and $|d| > 0.8$ = large effects. Significant *p*-values given in bold, italic

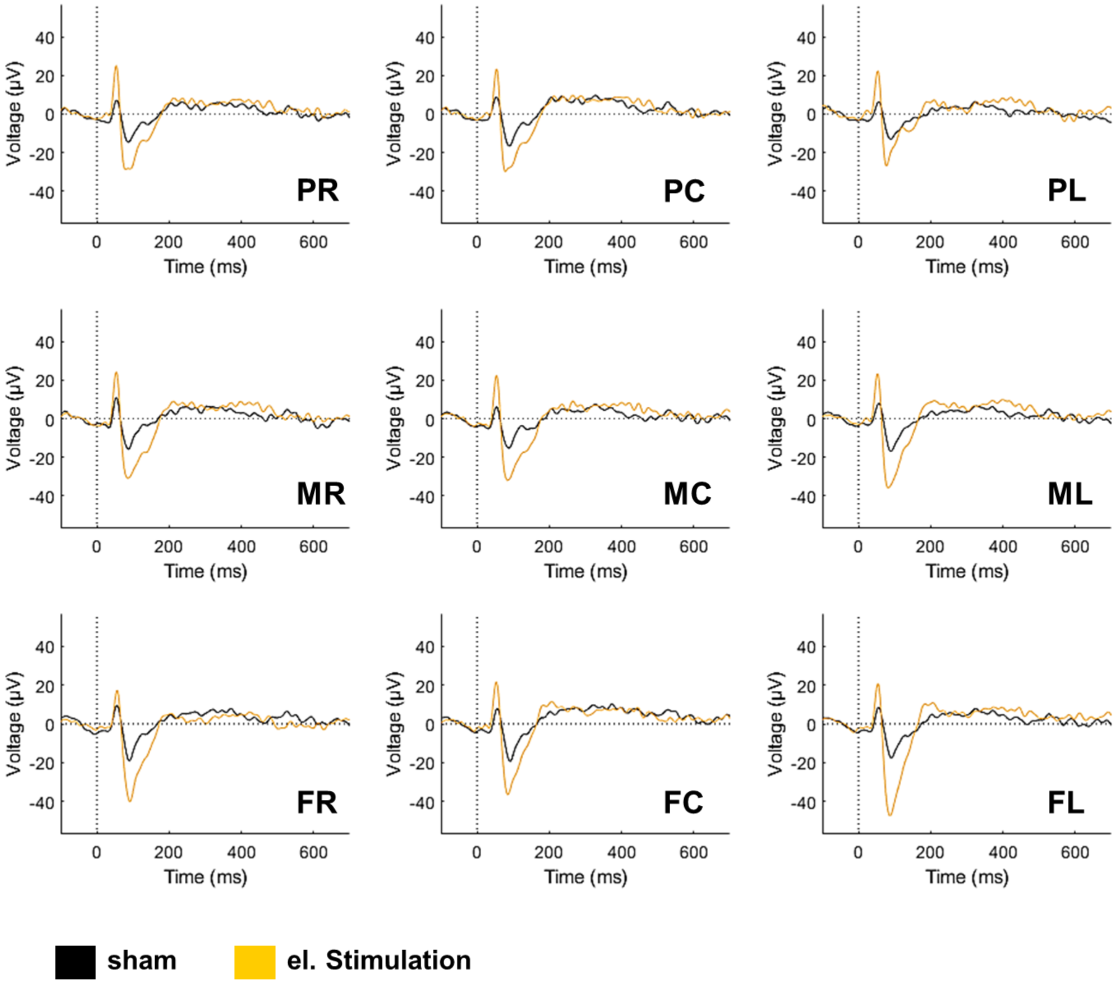

Figure S5 Grand average deviant-minus-standard ERP difference curves following electrical brain stimulation (n = 8, yellow) and untreated animals (sham, n = 10, black).

Table S8 Statistical analysis of ERP latencies following electrical brain stimulation

Electrode	ERP component	Comparison	Latency mean ± SEM (ms)			df	*t*	*p*	FDR-*p*	|*d*|
PR	P1	sham	57.167	±	2.374	5	1.688	0.152	0.351	0.689
		el. stim.	53.723	±	0.967					
	N1	sham	92.167	±	4.090	5	1.880	0.119	0.232	0.767
		el. stim.	85.278	±	2.361					
	P2	sham	121.390	±	2.429	5	-0.016	0.988	0.988	0.006
		el. stim.	121.445	±	1.746					
	N2	sham	145.388	±	3.373	5	2.151	0.084	0.284	0.878
		el. stim.	134.778	±	2.455					
	P3	sham	314.888	±	47.068	5	-0.239	0.820	0.934	0.098
		el. stim.	333.998	±	39.674					
MR	P1	sham	57.097	±	3.159	6	0.672	0.526	0.526	0.254
		el. stim.	54.857	±	1.217					
	N1	sham	94.999	±	3.942	6	0.891	0.407	0.458	0.337
		el. stim.	90.573	±	3.391					
	P2	sham	120.666	±	2.336	6	0.291	0.781	0.988	0.110
		el. stim.	119.476	±	2.555					
	N2	sham	149.237	±	4.980	6	2.016	0.090	0.284	0.762
		el. stim.	136.047	±	2.736					
	P3	sham	360.286	±	30.621	6	1.372	0.219	0.934	0.518
		el. stim.	294.380	±	26.758					
FR	P1	sham	61.001	±	3.019	6	1.847	0.114	0.351	0.698
		el. stim.	55.761	±	0.796					
	N1	sham	94.191	±	2.620	6	1.516	0.180	0.232	0.573
		el. stim.	88.856	±	2.404					
	P2	sham	119.904	±	2.155	6	-0.629	0.553	0.988	0.237
		el. stim.	121.429	±	2.108					
	N2	sham	137.571	±	4.172	6	0.456	0.665	0.665	0.172
		el. stim.	135.191	±	2.823					
	P3	sham	340.951	±	31.575	6	-0.184	0.860	0.934	0.069
		el. stim.	351.380	±	35.537					
FC	P1	sham	58.751	±	4.237	7	1.006	0.348	0.447	0.356
		el. stim.	54.915	±	1.113					
	N1	sham	95.791	±	2.592	7	1.846	0.107	0.232	0.653
		el. stim.	88.125	±	2.566					
	P2	sham	122.291	±	2.122	7	-0.044	0.966	0.966	0.016
		el. stim.	122.416	±	1.438					
	N2	sham	137.541	±	4.793	7	0.587	0.576	0.647	0.208
		el. stim.	134.124	±	2.388					
	P3	sham	355.666	±	31.867	7	0.416	0.690	0.934	0.147
		el. stim.	329.583	±	35.378					
PC	P1	sham	59.000	±	2.617	4	1.744	0.156	0.351	0.780
		el. stim.	55.266	±	0.938					
	N1	sham	95.266	±	4.248	4	2.024	0.113	0.232	0.905
		el. stim.	85.734	±	2.346					
	P2	sham	119.000	±	2.865	4	-0.122	0.908	0.988	0.055
		el. stim.	119.466	±	2.766					
	N2	sham	141.668	±	3.517	4	1.845	0.139	0.284	0.825
		el. stim.	135.000	±	2.231					
	P3	sham	272.668	±	29.917	4	-1.223	0.289	0.934	0.547
		el. stim.	354.468	±	38.430					

Table S8 Statistical analysis of ERP latencies following electrical brain stimulation (continued)

Electrode	ERP component	Comparison	Latency mean ± SEM (ms)			df	*t*	*p*	FDR-*p*	|*d*|
MC	P1	sham	57.476	±	2.932	6	1.020	0.347	0.447	0.386
		el. stim.	54.619	±	1.038					
	N1	sham	90.286	±	3.591	6	0.652	0.539	0.539	0.246
		el. stim.	87.237	±	2.641					
	P2	sham	121.429	±	1.770	6	-0.901	0.402	0.988	0.341
		el. stim.	123.333	±	1.077					
	N2	sham	146.856	±	5.528	6	1.888	0.108	0.284	0.713
		el. stim.	135.381	±	2.376					
	P3	sham	345.953	±	37.716	6	0.296	0.777	0.934	0.112
		el. stim.	330.000	±	33.545					
FL	P1	sham	58.081	±	4.222	7	0.891	0.402	0.453	0.315
		el. stim.	54.664	±	0.960					
	N1	sham	96.084	±	2.758	7	2.403	***0.047***	0.232	0.849
		el. stim.	87.875	±	2.124					
	P2	sham	121.750	±	2.505	7	-0.668	0.525	0.942	0.236
		el. stim.	123.543	±	0.959					
	N2	sham	144.000	±	5.206	7	1.167	0.282	0.422	0.412
		el. stim.	136.125	±	2.621					
	P3	sham	334.668	±	34.762	7	0.542	0.604	0.934	0.192
		el. stim.	302.750	±	36.122					
ML	P1	sham	59.570	±	2.848	6	1.658	0.148	0.351	0.627
		el. stim.	54.474	±	1.262					
	N1	sham	94.619	±	3.035	6	1.519	0.180	0.232	0.574
		el. stim.	88.333	±	2.577					
	P2	sham	123.096	±	1.231	6	-0.694	0.514	0.988	0.262
		el. stim.	123.237	±	1.143					
	N2	sham	147.334	±	6.126	6	1.612	0.158	0.284	0.609
		el. stim.	136.953	±	2.823					
	P3	sham	350.763	±	36.646	6	0.087	0.934	0.934	0.033
		el. stim.	345.856	±	37.992					
PL	P1	sham	57.280	±	1.676	5	1.129	0.310	0.447	0.461
		el. stim.	54.945	±	1.361					
	N1	sham	95.055	±	3.450	5	1.735	0.143	0.232	0.708
		el. stim.	85.943	±	2.735					
	P2	sham	122.390	±	1.456	5	0.408	0.700	0.988	0.167
		el. stim.	121.943	±	1.272					
	N2	sham	150.943	±	6.512	5	0.773	0.474	0.610	0.316
		el. stim.	141.778	±	6.389					
	P3	sham	334.887	±	28.658	5	0.317	0.764	0.934	0.129
		el. stim.	319.443	±	42.447					

SEM: standard error of the mean, df: degrees of freedom related to available channel data, FDR-*p*: false discovery rate-adjusted *p*-value, $|d|$: effect size Cohen´s *d* with $|d| \geq 0.2$ = small, $|d| \geq 0.5$ = medium and $|d| > 0.8$ = large effects. Significant *p*-values given in bold, italic

Table S9 Statistical analysis of ERP amplitudes following electrical brain stimulation

Electrode	ERP component	Comparison	Amplitude mean ± SEM (µV)			df	*t*	*p*	FDR-*p*	|*d*|
PR	P1	sham	7.342	±	3.567	5	-2.033	0.098	0.144	0.830
		el. stim.	22.083	±	4.890					
	N1	sham	-17.690	±	6.009	5	2.103	0.089	0.100	0.859
		el. stim.	-32.360	±	3.747					
	P2	sham	15.037	±	7.294	5	-0.921	0.399	0.513	0.376
		el. stim.	19.507	±	5.044					
	N2	sham	-6.808	±	3.824	5	1.746	0.141	0.182	0.713
		el. stim.	-14.535	±	2.076					
	P3	sham	12.700	±	3.875	5	-1.072	0.333	0.915	0.438
		el. stim.	15.572	±	2.781					
MR	P1	sham	8.026	±	2.471	6	-2.170	0.073	0.144	0.820
		el. stim.	22.020	±	4.623					
	N1	sham	-15.791	±	6.966	6	2.638	***0.039***	0.058	0.997
		el. stim.	-37.571	±	6.292					
	P2	sham	16.519	±	7.921	6	-0.128	0.902	0.902	0.048
		el. stim.	17.031	±	5.745					
	N2	sham	-5.959	±	3.080	6	2.268	0.064	0.103	0.857
		el. stim.	-20.431	±	4.776					
	P3	sham	13.309	±	5.207	6	-0.954	0.377	0.915	0.361
		el. stim.	17.726	±	2.361					
FR	P1	sham	9.743	±	2.522	6	-1.052	0.333	0.333	0.398
		el. stim.	14.617	±	4.059					
	N1	sham	-19.927	±	7.506	6	2.699	***0.036***	0.058	1.020
		el. stim.	-41.386	±	6.996					
	P2	sham	14.510	±	8.225	6	-0.965	0.372	0.513	0.365
		el. stim.	18.699	±	5.500					
	N2	sham	-6.150	±	3.177	6	3.527	***0.012***	***0.056***	1.333
		el. stim.	-22.513	±	6.126					
	P3	sham	16.916	±	5.443	6	0.548	0.604	0.915	0.207
		el. stim.	14.316	±	3.773					
FC	P1	sham	7.786	±	4.196	7	-1.815	0.112	0.144	0.642
		el. stim.	18.958	±	4.295					
	N1	sham	-19.060	±	6.982	7	2.692	***0.031***	0.058	0.952
		el. stim.	-40.701	±	5.122					
	P2	sham	14.535	±	7.229	7	-1.030	0.337	0.513	0.365
		el. stim.	22.194	±	6.413					
	N2	sham	-5.291	±	5.596	7	2.149	0.069	0.103	0.760
		el. stim.	-16.404	±	4.702					
	P3	sham	19.374	±	5.176	7	0.280	0.788	0.915	0.099
		el. stim.	18.280	±	3.679					
PC	P1	sham	11.130	±	3.049	4	-1.191	0.299	0.333	0.533
		el. stim.	18.544	±	4.418					
	N1	sham	-17.108	±	6.496	4	2.128	0.100	0.100	0.952
		el. stim.	-28.642	±	5.304					
	P2	sham	12.662	±	6.587	4	-0.474	0.660	0.743	0.212
		el. stim.	15.036	±	4.626					
	N2	sham	-8.686	±	4.036	4	1.596	0.186	0.209	0.714
		el. stim.	-17.644	±	3.912					
	P3	sham	16.094	±	2.862	4	-0.252	0.814	0.915	0.113
		el. stim.	16.846	±	2.941					

Table S9 Statistical analysis of ERP amplitudes following electrical brain stimulation (continued)

Electrode	ERP component	Comparison	Amplitude mean ± SEM (µV)			df	*t*	*p*	FDR-*p*	$\lvert d \rvert$
MC	P1	sham	5.717	±	3.144	6	-2.084	0.082	0.144	0.788
		el. stim.	19.059	±	4.435					
	N1	sham	-14.830	±	5.458	6	3.664	***0.011***	***0.047***	1.385
		el. stim.	-38.593	±	6.313					
	P2	sham	14.036	±	6.971	6	-1.128	0.302	0.513	0.426
		el. stim.	18.933	±	5.415					
	N2	sham	-6.336	±	4.662	6	2.590	***0.041***	0.093	0.979
		el. stim.	-19.926	±	4.432					
	P3	sham	16.697	±	4.329	6	-0.088	0.933	0.933	0.033
		el. stim.	17.054	±	2.377					
FL	P1	sham	6.724	±	2.438	7	-2.449	***0.044***	0.144	0.866
		el. stim.	17.919	±	3.322					
	N1	sham	-15.875	±	4.418	7	3.772	***0.007***	***0.047***	1.334
		el. stim.	-53.006	±	8.670					
	P2	sham	11.875	±	6.150	7	-2.101	0.074	0.441	0.742
		el. stim.	26.773	±	5.602					
	N2	sham	-6.061	±	3.242	7	3.439	***0.011***	0.056	1.216
		el. stim.	-22.410	±	5.350					
	P3	sham	16.400	±	3.607	7	-0.404	0.699	0.915	0.143
		el. stim.	17.888	±	2.297					
ML	P1	sham	6.850	±	2.475	6	-2.071	0.084	0.144	0.783
		el. stim.	21.147	±	4.957					
	N1	sham	-19.130	±	6.999	6	2.764	***0.033***	0.058	1.045
		el. stim.	-46.196	±	8.656					
	P2	sham	14.774	±	7.170	6	-1.255	0.256	0.513	0.474
		el. stim.	27.226	±	8.243					
	N2	sham	-7.496	±	2.905	6	2.890	***0.028***	0.083	1.092
		el. stim.	-17.449	±	4.522					
	P3	sham	12.463	±	4.910	6	-1.149	0.294	0.915	0.434
		el. stim.	17.934	±	3.026					
PL	P1	sham	6.195	±	2.913	5	-1.966	0.106	0.144	0.803
		el. stim.	20.253	±	5.263					
	N1	sham	-13.268	±	4.960	5	2.015	0.100	0.100	0.822
		el. stim.	-31.863	±	6.167					
	P2	sham	11.030	±	3.312	5	-2.031	0.098	0.441	0.829
		el. stim.	23.905	±	6.201					
	N2	sham	-6.713	±	3.227	5	1.027	0.351	0.351	0.419
		el. stim.	-11.150	±	3.582					
	P3	sham	9.882	±	3.199	5	-0.821	0.449	0.915	0.335
		el. stim.	14.470	±	4.927					

SEM: standard error of the mean, df: degrees of freedom related to available channel data, FDR-*p*: false discovery rate-adjusted *p*-value, $|d|$: effect size Cohen´s *d* with $|d| \geq 0.2$ = small, $|d| \geq 0.5$ = medium and $|d| > 0.8$ = large effects. Significant *p*-values given in bold, italic

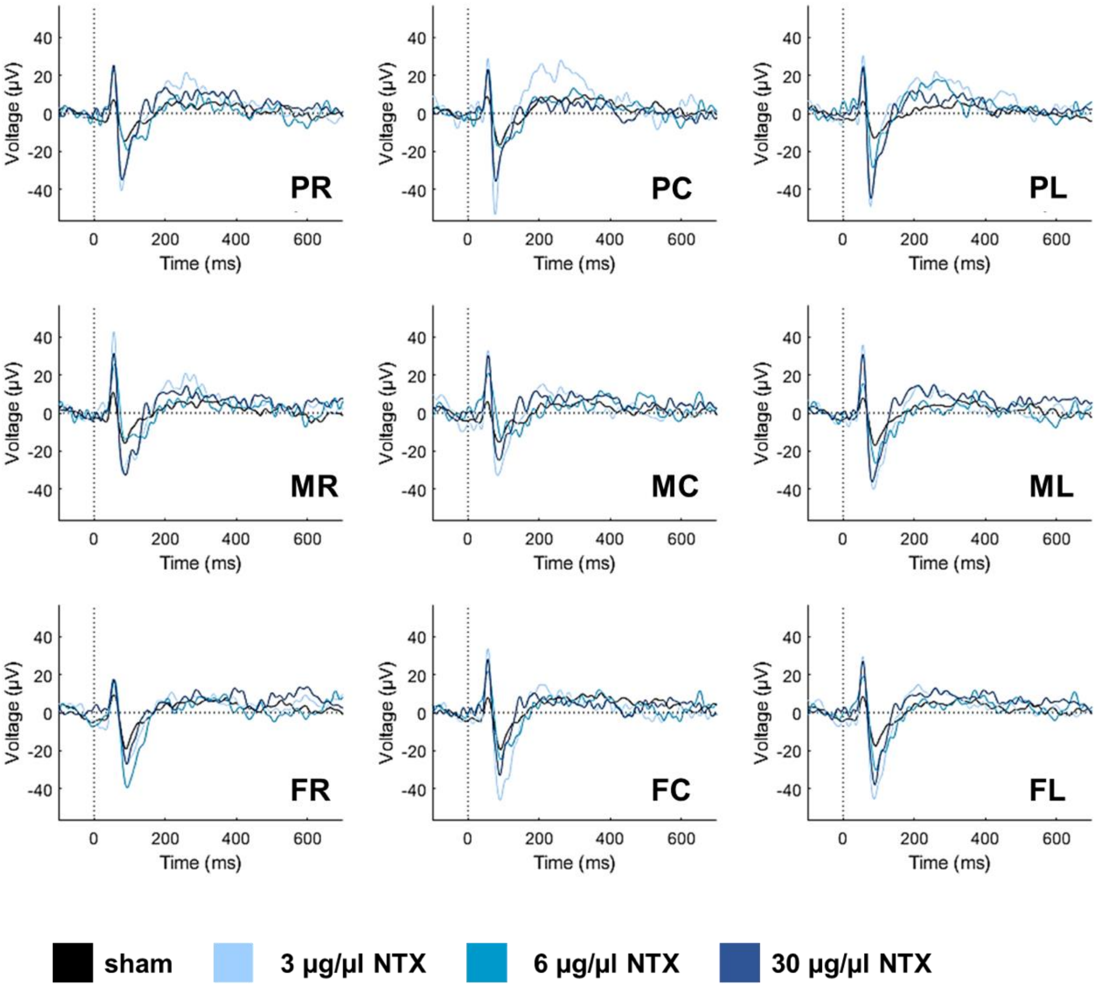

Figure S6 Grand average deviant-minus-standard ERP difference curves following cortical delivery of naltrexone (NTX) at 3 μg/μl (n = 5, light blue), 6 μg/μl (n = 4, mid-blue) and 30 μg/μl (n = 5, dark blue) and untreated animals (sham, n = 10, black).

Table S10 Statistical analysis of ERP latencies following NTX administration at 3 µg/µl

Electrode	ERP component	Comparison	Latency mean ± SEM (ms)			df	*t*	*p*	FDR-*p*	|*d*|
PR	P1	sham	60.110	±	2.792	2	1.388	0.300	0.748	0.801
		NTX3	54.887	±	0.987					
	N1	sham	95.557	±	7.838	2	1.990	0.185	0.259	1.149
		NTX3	80.223	±	0.223					
	P2	sham	120.000	±	5.000	2	-0.016	0.423	0.511	0.577
		NTX3	125.000	±	0.000					
	N2	sham	146.443	±	6.951	2	1.160	0.366	0.585	0.670
		NTX3	135.110	±	5.110					
	P3	sham	325.557	±	53.964	2	0.630	0.593	0.967	0.363
		NTX3	270.777	±	36.381					
MR	P1	sham	60.333	±	7.396	2	0.596	0.611	0.748	0.344
		NTX3	55.330	±	1.000					
	N1	sham	105.000	±	0.000	2	3.156	0.087	0.204	1.822
		NTX3	89.667	±	4.859					
	P2	sham	120.000	±	5.000	2	-0.068	0.952	0.952	0.039
		NTX3	120.557	±	4.443					
	N2	sham	154.667	±	8.759	2	1.446	0.285	0.570	0.835
		NTX3	135.000	±	5.000					
	P3	sham	352.110	±	44.936	2	0.974	0.433	0.967	0.563
		NTX3	270.333	±	39.306					
FR	P1	sham	66.585	±	2.579	3	4.827	***0.017***	0.136	2.413
		NTX3	55.750	±	1.141					
	N1	sham	98.668	±	2.241	3	1.428	0.249	0.290	0.714
		NTX3	91.003	±	3.274					
	P2	sham	117.250	±	3.092	3	-2.007	0.138	0.511	1.003
		NTX3	123.668	±	1.333					
	N2	sham	137.583	±	7.583	3	-0.322	0.769	0.769	0.161
		NTX3	142.085	±	10.282					
	P3	sham	315.833	±	21.008	3	-0.034	0.975	0.975	0.017
		NTX3	317.418	±	36.750					
FC	P1	sham	58.918	±	8.249	3	0.418	0.704	0.748	0.209
		NTX3	55.168	±	1.288					
	N1	sham	98.333	±	2.691	3	2.137	0.122	0.214	1.068
		NTX3	91.083	±	1.423					
	P2	sham	120.750	±	4.250	3	-1.000	0.391	0.493	0.500
		NTX3	125.000	±	0.000					
	N2	sham	143.083	±	9.193	3	0.420	0.703	0.769	0.210
		NTX3	137.168	±	7.168					
	P3	sham	314.833	±	49.015	3	0.386	0.725	0.967	0.193
		NTX3	288.003	±	47.933					
PC	P1	sham	62.330	±	.	0	.	.	.	.
		NTX3	56.000	±	.					
	N1	sham	105.000	±	.	0	.	.	.	.
		NTX3	80.000	±	.					
	P2	sham	125.000	±	.	0	.	.	.	.
		NTX3	125.000	±	.					
	N2	sham	143.000	±	.	0	.	.	.	.
		NTX3	144.330	±	.					
	P3	sham	280.000	±	.	0	.	.	.	.
		NTX3	330.000	±	.					

Table S10 Statistical analysis of ERP latencies following NTX administration at 3 µg/µl (continued)

Electrode	ERP component	Comparison	Latency mean ± SEM (ms)			df	*t*	*p*	FDR-*p*	|*d*|
MC	P1	sham	59.665	±	5.130	3	0.711	0.528	0.748	0.356
		NTX3	56.000	±	0.934					
	N1	sham	93.500	±	5.158	3	1.279	0.291	0.291	0.640
		NTX3	86.333	±	3.554					
	P2	sham	123.333	±	1.668	3	-1.000	0.391	0.511	0.641
		NTX3	124.418	±	0.583					
	N2	sham	151.915	±	8.643	3	0.781	0.492	0.656	0.391
		NTX3	141.918	±	5.569					
	P3	sham	328.335	±	43.903	3	1.419	0.251	0.967	0.710
		NTX3	240.418	±	32.271					
FL	P1	sham	59.998	±	6.887	4	0.685	0.531	0.748	0.306
		NTX3	54.934	±	0.891					
	N1	sham	100.534	±	2.370	4	3.224	***0.032***	0.114	1.442
		NTX3	87.734	±	2.225					
	P2	sham	122.000	±	3.000	4	-0.860	0.438	0.511	0.384
		NTX3	124.666	±	0.334					
	N2	sham	147.400	±	8.009	4	1.812	0.144	0.385	0.810
		NTX3	132.000	±	1.378					
	P3	sham	311.934	±	39.098	4	-0.063	0.952	0.975	0.028
		NTX3	315.868	±	44.399					
ML	P1	sham	63.583	±	3.905	3	2.146	0.121	0.484	1.073
		NTX3	54.750	±	1.151					
	N1	sham	99.583	±	3.146	3	3.772	***0.033***	0.114	1.886
		NTX3	84.585	±	2.367					
	P2	sham	123.250	±	1.750	3	-1.000	0.391	0.511	0.500
		NTX3	125.000	±	0.000					
	N2	sham	155.085	±	8.495	3	2.861	0.065	0.385	1.431
		NTX3	136.000	±	3.830					
	P3	sham	337.253	±	41.296	3	0.530	0.633	0.967	0.265
		NTX3	291.668	±	46.632					
PL	P1	sham	57.835	±	3.165	1	0.418	0.748	0.748	0.295
		NTX3	56.165	±	0.835					
	N1	sham	105.000	±	0.000	1	.	.	.	.
		NTX3	80.000	±	0.000					
	P2	sham	125.000	±	0.000	1	.	.	.	.
		NTX3	125.000	±	0.000					
	N2	sham	165.665	±	2.335	1	4.555	0.138	0.385	3.221
		NTX3	138.335	±	8.335					
	P3	sham	358.000	±	80.330	1	0.543	0.683	0.967	0.384
		NTX3	276.835	±	69.165					

SEM: standard error of the mean, df: degrees of freedom related to available channel data, FDR-*p*: false discovery rate-adjusted p-value, |*d*|: effect size Cohen´s *d* with $|d| \geq 0.2$ = small, $|d| \geq 0.5$ = medium and $|d| > 0.8$ = large effects. Significant *p*-values given in bold, italic

Table S11 Statistical analysis of ERP amplitudes following NTX administration at 3 µg/µl

Electrode	ERP component	Comparison	Amplitude mean ± SEM (µV)			df	*t*	*p*	FDR-*p*	|*d*|
PR	P1	sham	3.860	±	5.858	2	-1.537	0.264	0.434	0.887
		NTX3	19.023	±	5.151					
	N1	sham	-16.343	±	5.430	2	2.036	0.179	0.204	1.175
		NTX3	-40.203	±	8.066					
	P2	sham	6.810	±	3.942	2	-1.604	0.250	0.286	0.926
		NTX3	21.910	±	10.520					
	N2	sham	-12.470	±	3.295	2	0.321	0.779	0.779	0.185
		NTX3	-14.027	±	5.350					
	P3	sham	7.657	±	2.656	2	-1.971	0.187	0.404	1.138
		NTX3	24.367	±	6.610					
MR	P1	sham	4.927	±	3.186	2	-1.751	0.222	0.434	1.011
		NTX3	34.920	±	14.168					
	N1	sham	-3.553	±	6.456	2	1.818	0.211	0.211	1.050
		NTX3	-29.947	±	11.270					
	P2	sham	5.907	±	1.186	2	-0.745	0.534	0.534	0.430
		NTX3	9.957	±	5.253					
	N2	sham	-9.883	±	3.573	2	4.684	***0.043***	0.184	2.704
		NTX3	-20.083	±	4.946					
	P3	sham	8.093	±	2.915	2	-1.784	0.216	0.404	1.030
		NTX3	24.677	±	7.289					
FR	P1	sham	7.293	±	3.203	3	-0.901	0.434	0.434	0.450
		NTX3	13.418	±	5.408					
	N1	sham	-5.380	±	1.803	3	1.803	0.169	0.204	0.901
		NTX3	-24.980	±	12.039					
	P2	sham	1.898	±	1.316	3	-5.005	***0.015***	0.050	2.502
		NTX3	9.523	±	1.487					
	N2	sham	-5.188	±	2.672	3	1.465	0.239	0.319	0.732
		NTX3	-14.505	±	8.128					
	P3	sham	9.093	±	1.707	3	-1.168	0.327	0.404	0.584
		NTX3	15.143	±	6.756					
FC	P1	sham	9.703	±	6.952	3	-0.979	0.400	0.434	0.490
		NTX3	27.773	±	12.020					
	N1	sham	-5.463	±	3.782	3	5.529	***0.012***	***0.031***	2.765
		NTX3	-43.965	±	6.174					
	P2	sham	5.283	±	4.060	3	-5.035	***0.015***	0.050	2.517
		NTX3	15.130	±	5.243					
	N2	sham	-2.075	±	8.891	3	3.085	0.054	0.184	1.543
		NTX3	-22.330	±	5.505					
	P3	sham	16.158	±	6.975	3	-0.488	0.659	0.659	0.244
		NTX3	21.118	±	4.415					
PC	P1	sham	2.380	±	.	0	.	.	.	.
		NTX3	5.740	±	.					
	N1	sham	-12.760	±	.	0	.	.	.	.
		NTX3	-25.110	±	.					
	P2	sham	-12.240	±	.	0	.	.	.	.
		NTX3	-0.410	±	.					
	N2	sham	-6.360	±	.	0	.	.	.	.
		NTX3	-10.310	±	.					
	P3	sham	9.930	±	.	0	.	.	.	.
		NTX3	19.540	±	.					

Table S11 Statistical analysis of ERP amplitudes following NTX administration at 3 µg/µl (continued)

Electrode	ERP component	Comparison	Amplitude mean ± SEM (µV)			df	*t*	*p*	FDR-*p*	|*d*|
MC	P1	sham	2.543	±	4.117	3	-1.235	0.305	0.434	0.618
		NTX3	24.825	±	14.421					
	N1	sham	-4.938	±	2.002	3	4.223	***0.024***	***0.049***	2.112
		NTX3	-34.480	±	7.293					
	P2	sham	4.840	±	1.768	3	-3.119	0.053	0.071	1.560
		NTX3	22.528	±	7.245					
	N2	sham	-4.323	±	4.856	3	2.074	0.130	0.208	1.037
		NTX3	-9.500	±	4.217					
	P3	sham	11.348	±	2.828	3	-1.585	0.211	0.404	0.792
		NTX3	22.463	±	4.703					
FL	P1	sham	4.992	±	3.581	4	-1.262	0.276	0.434	0.564
		NTX3	23.008	±	12.619					
	N1	sham	-8.520	±	1.676	4	9.094	***0.001***	***0.006***	4.067
		NTX3	-44.646	±	3.501					
	P2	sham	4.274	±	1.874	4	-3.512	***0.025***	0.050	1.571
		NTX3	28.846	±	7.490					
	N2	sham	-5.342	±	4.224	4	2.470	0.069	0.184	1.105
		NTX3	-15.306	±	4.896					
	P3	sham	11.868	±	1.930	4	-1.048	0.354	0.404	0.469
		NTX3	18.932	±	5.196					
ML	P1	sham	4.148	±	3.027	3	-1.460	0.240	0.434	0.730
		NTX3	29.165	±	14.356					
	N1	sham	-7.265	±	1.775	3	7.309	***0.005***	***0.021***	3.654
		NTX3	-38.913	±	4.627					
	P2	sham	4.180	±	1.272	3	-3.514	***0.039***	0.062	1.757
		NTX3	20.008	±	4.758					
	N2	sham	-7.925	±	3.222	3	2.374	0.098	0.196	1.187
		NTX3	-16.000	±	3.910					
	P3	sham	7.593	±	1.682	3	-1.729	0.182	0.404	0.864
		NTX3	19.920	±	6.541					
PL	P1	sham	-0.220	±	3.350	1	-1.241	0.432	0.434	0.878
		NTX3	21.835	±	14.415					
	N1	sham	-5.195	±	3.185	1	12.282	0.052	0.083	8.685
		NTX3	-48.120	±	6.680					
	P2	sham	5.095	±	0.095	1	-27.997	***0.023***	0.050	19.797
		NTX3	47.230	±	1.600					
	N2	sham	-9.160	±	8.550	1	-0.541	0.684	0.779	0.383
		NTX3	-3.030	±	2.780					
	P3	sham	6.795	±	3.235	1	-2.317	0.259	0.404	1.638
		NTX3	26.570	±	5.300					

SEM: standard error of the mean, df: degrees of freedom related to available channel data, FDR-*p*: false discovery rate-adjusted *p*-value, |*d*|: effect size Cohen´s *d* with $|d| \geq 0.2$ = small, $|d| \geq 0.5$ = medium and $|d| > 0.8$ = large effects. Significant *p*-values given in bold, italic

Table S12 Statistical analysis of ERP latencies following NTX administration at 6 μg/μl

Electrode	ERP component	Comparison	Latency mean	±	SEM (ms)	df	*t*	*p*	FDR-*p*	|*d*|
PR	P1	sham	60.250	±	1.979	3	1.035	0.377	0.675	0.518
		NTX6	56.583	±	1.888					
	N1	sham	94.918	±	5.579	3	0.683	0.544	0.544	0.342
		NTX6	91.083	±	3.185					
	P2	sham	121.250	±	3.750	3	1.000	0.391	0.750	0.500
		NTX6	117.500	±	4.330					
	N2	sham	146.333	±	4.916	3	1.228	0.307	0.491	0.614
		NTX6	142.585	±	4.320					
	P3	sham	300.250	±	45.787	3	0.474	0.668	0.902	0.237
		NTX6	277.165	±	36.554					
MR	P1	sham	60.333	±	7.396	2	0.236	0.835	0.923	0.136
		NTX6	58.337	±	3.283					
	N1	sham	105.000	±	0.000	2	3.296	0.081	0.544	1.903
		NTX6	90.000	±	4.550					
	P2	sham	120.000	±	5.000	2	0.013	0.991	0.991	0.007
		NTX6	119.890	±	4.946					
	N2	sham	154.667	±	8.759	2	1.527	0.266	0.491	0.882
		NTX6	134.557	±	4.557					
	P3	sham	352.110	±	44.936	2	0.515	0.658	0.902	0.297
		NTX6	314.447	±	28.910					
FR	P1	sham	69.000	±	4.000	1	3.822	0.163	0.652	2.702
		NTX6	58.165	±	1.165					
	N1	sham	98.170	±	2.500	1	0.883	0.539	0.544	0.625
		NTX6	93.165	±	3.165					
	P2	sham	117.500	±	7.500	1	-1.000	0.500	0.750	0.707
		NTX6	125.000	±	0.000					
	N2	sham	130.000	±	0.000	1	-1.000	0.500	0.525	0.707
		NTX6	135.500	±	5.500					
	P3	sham	312.000	±	32.330	1	0.219	0.863	0.902	0.155
		NTX6	287.835	±	77.835					
FC	P1	sham	58.918	±	8.249	3	0.225	0.837	0.923	0.112
		NTX6	56.833	±	1.985					
	N1	sham	98.333	±	2.691	3	1.128	0.342	0.544	0.564
		NTX6	95.418	±	3.337					
	P2	sham	120.750	±	4.250	3	0.427	0.698	0.785	0.213
		NTX6	117.500	±	4.330					
	N2	sham	143.083	±	9.193	3	0.718	0.525	0.525	0.359
		NTX6	136.250	±	3.789					
	P3	sham	314.833	±	49.015	3	-0.134	0.902	0.902	0.067
		NTX6	319.083	±	35.247					
PC	P1	sham	63.165	±	0.835	1	6.410	0.099	0.652	4.533
		NTX6	55.665	±	0.335					
	N1	sham	105.000	±	0.000	1	1.818	0.320	0.544	1.286
		NTX6	91.665	±	7.335					
	P2	sham	117.500	±	7.500	1	-1.000	0.500	0.750	0.707
		NTX6	125.000	±	0.000					
	N2	sham	136.500	±	6.500	1	-1.000	0.500	0.525	0.707
		NTX6	138.665	±	8.665					
	P3	sham	312.835	±	32.835	1	0.341	0.791	0.902	0.241
		NTX6	298.500	±	9.170					

Table S12 Statistical analysis of ERP latencies following NTX administration at 6 µg/µl (continued)

Electrode	ERP component	Comparison	Latency mean ± SEM (ms)			df	*t*	*p*	FDR-*p*	|*d*|
MC	P1	sham	63.553	±	4.732	2	1.002	0.422	0.675	0.578
		NTX6	57.557	±	3.664					
	N1	sham	98.000	±	3.565	2	0.920	0.455	0.544	0.531
		NTX6	95.557	±	1.456					
	P2	sham	125.000	±	0.000	2	2.000	0.184	0.750	1.155
		NTX6	115.000	±	5.000					
	N2	sham	159.220	±	6.532	2	2.198	0.159	0.490	1.269
		NTX6	139.223	±	4.660					
	P3	sham	358.557	±	45.037	2	0.483	0.677	0.902	0.279
		NTX6	327.667	±	49.960					
FL	P1	sham	57.915	±	8.475	3	0.105	0.923	0.923	0.052
		NTX6	56.835	±	2.518					
	N1	sham	99.918	±	2.954	3	1.335	0.274	0.544	0.667
		NTX6	95.168	±	3.373					
	P2	sham	121.250	±	3.750	3	0.522	0.638	0.785	0.261
		NTX6	117.500	±	4.330					
	N2	sham	151.750	±	8.681	3	1.721	0.184	0.490	0.861
		NTX6	139.168	±	3.329					
	P3	sham	311.085	±	50.464	3	0.283	0.796	0.902	0.141
		NTX6	299.418	±	47.119					
ML	P1	sham	63.583	±	3.905	3	1.375	0.263	0.675	0.688
		NTX6	56.500	±	2.331					
	N1	sham	99.583	±	3.146	3	1.709	0.186	0.544	0.854
		NTX6	90.750	±	2.146					
	P2	sham	123.250	±	1.750	3	1.591	0.210	0.750	0.795
		NTX6	117.500	±	4.330					
	N2	sham	155.085	±	8.495	3	2.677	0.075	0.490	1.339
		NTX6	137.333	±	4.242					
	P3	sham	337.253	±	41.296	3	-0.558	0.616	0.902	0.279
		NTX6	354.000	±	20.012					
PL	P1	sham	60.253	±	2.790	3	1.379	0.262	0.441	0.689
		NTX6	50.080	±	4.679					
	N1	sham	100.665	±	3.799	3	2.623	0.079	0.108	1.311
		NTX6	85.500	±	3.634					
	P2	sham	125.000	±	0.000	3	1.539	0.221	0.842	0.770
		NTX6	119.750	±	3.411					
	N2	sham	147.833	±	10.340	3	1.197	0.317	0.792	0.599
		NTX6	138.250	±	8.250					
	P3	sham	315.750	±	41.082	3	0.548	0.622	0.822	0.274
		NTX6	277.500	±	42.430					

SEM: standard error of the mean, df: degrees of freedom related to available channel data, FDR-*p*: false discovery rate-adjusted *p*-value, $|d|$: effect size Cohen´s *d* with $|d| \geq 0.2$ = small, $|d| \geq 0.5$ = medium and $|d| > 0.8$ = large effects. Significant *p*-values given in bold, italic

Table S13 Statistical analysis of ERP amplitudes following NTX administration at 6 µg/µl

Electrode	ERP component	Comparison	Amplitude mean ± SEM (µV)			df	*t*	*p*	FDR-*p*	|*d*|
PR	P1	sham	5.813	±	4.579	3	-2.861	0.065	0.280	1.431
		NTX6	20.113	±	0.702					
	N1	sham	-12.033	±	5.773	3	1.195	0.318	0.440	0.598
		NTX6	-18.825	±	5.187					
	P2	sham	7.743	±	2.939	3	-0.558	0.616	0.616	0.279
		NTX6	9.645	±	3.174					
	N2	sham	-7.188	±	5.774	3	1.192	0.319	0.365	0.596
		NTX6	-15.108	±	1.945					
	P3	sham	10.143	±	3.115	3	-2.512	0.087	0.174	1.256
		NTX6	15.980	±	2.790					
MR	P1	sham	4.927	±	3.186	2	-3.346	0.079	0.280	1.932
		NTX6	25.100	±	3.251					
	N1	sham	-3.553	±	6.456	2	0.629	0.594	0.594	0.363
		NTX6	-13.183	±	10.765					
	P2	sham	5.907	±	1.186	2	0.260	0.532	0.599	0.432
		NTX6	9.047	±	4.550					
	N2	sham	-9.883	±	3.573	2	5.014	***0.038***	0.098	2.895
		NTX6	-16.690	±	4.818					
	P3	sham	8.093	±	2.915	2	-2.739	0.111	0.178	1.582
		NTX6	14.523	±	2.020					
FR	P1	sham	10.890	±	2.980	1	-1.626	0.351	0.351	1.150
		NTX6	14.475	±	0.775					
	N1	sham	-7.890	±	2.390	1	4.504	0.139	0.371	3.185
		NTX6	-38.200	±	9.120					
	P2	sham	0.765	±	0.185	1	-7.030	0.090	0.400	4.971
		NTX6	14.615	±	1.785					
	N2	sham	-7.830	±	5.240	1	12.970	***0.049***	0.098	9.171
		NTX6	-20.995	±	6.255					
	P3	sham	10.575	±	0.695	1	-1.988	0.297	0.396	1.406
		NTX6	12.285	±	1.555					
FC	P1	sham	9.703	±	6.952	3	-1.114	0.346	0.351	0.557
		NTX6	20.703	±	4.598					
	N1	sham	-5.463	±	3.782	3	1.606	0.207	0.413	0.803
		NTX6	-23.173	±	10.597					
	P2	sham	5.283	±	4.060	3	-1.578	0.213	0.400	0.789
		NTX6	11.955	±	3.333					
	N2	sham	-2.075	±	8.891	3	3.939	***0.029***	0.098	1.970
		NTX6	-16.710	±	6.080					
	P3	sham	16.158	±	6.975	3	-0.111	0.918	0.918	0.056
		NTX6	16.775	±	2.046					
PC	P1	sham	7.200	±	4.820	1	-2.524	0.240	0.320	-1.785
		NTX6	16.060	±	1.310					
	N1	sham	-12.575	±	0.185	1	1.753	0.330	0.440	1.240
		NTX6	-26.310	±	7.650					
	P2	sham	0.525	±	0.005	1	-1.422	0.390	0.501	1.006
		NTX6	13.085	±	8.835					
	N2	sham	-10.300	±	3.940	1	0.417	0.748	0.748	0.295
		NTX6	-12.875	±	2.235					
	P3	sham	11.150	±	1.220	1	-1.104	0.469	0.535	-0.781
		NTX6	13.910	±	1.280					

Table S13 Statistical analysis of ERP amplitudes following NTX administration at 6 µg/µl (continued)

Electrode	ERP component	Comparison	Amplitude mean ± SEM (µV)			df	*t*	*p*	FDR-*p*	|*d*|
MC	P1	sham	2.803	±	5.811	2	-1.957	0.189	0.303	1.130
		NTX6	22.050	±	4.386					
	N1	sham	-6.333	±	2.031	2	0.911	0.458	0.524	0.526
		NTX6	-12.233	±	4.506					
	P2	sham	3.770	±	1.991	2	-1.523	0.267	0.401	0.879
		NTX6	8.887	±	4.308					
	N2	sham	-8.287	±	3.967	2	1.703	0.231	0.308	0.983
		NTX6	-12.563	±	5.094					
	P3	sham	8.820	±	1.795	2	-4.156	0.053	0.142	2.400
		NTX6	16.567	±	1.397					
FL	P1	sham	6.888	±	3.923	3	-1.845	0.162	0.303	0.923
		NTX6	19.075	±	3.619					
	N1	sham	-8.485	±	2.163	3	2.142	0.122	0.371	1.071
		NTX6	-28.350	±	9.616					
	P2	sham	3.773	±	2.332	3	-3.527	***0.039***	0.351	1.763
		NTX6	14.850	±	2.391					
	N2	sham	-6.343	±	5.298	3	3.414	***0.042***	0.098	1.707
		NTX6	-16.173	±	5.697					
	P3	sham	10.235	±	1.327	3	-3.987	***0.028***	0.142	1.993
		NTX6	15.295	±	2.021					
ML	P1	sham	4.148	±	3.027	3	-2.302	0.105	0.280	1.151
		NTX6	15.178	±	1.835					
	N1	sham	-7.265	±	1.775	3	2.776	0.069	0.371	1.388
		NTX6	-25.013	±	6.348					
	P2	sham	4.180	±	1.272	3	-1.621	0.203	0.400	0.811
		NTX6	12.923	±	4.447					
	N2	sham	-7.925	±	3.222	3	2.898	0.063	0.100	1.449
		NTX6	-15.735	±	1.032					
	P3	sham	7.593	±	1.682	3	-3.430	***0.042***	0.142	1.715
		NTX6	14.348	±	1.064					
PL	P1	sham	1.850	±	5.420	1	-1.370	0.401	0.401	0.968
		NTX6	22.155	±	9.405					
	N1	sham	-15.990	±	7.610	1	0.877	0.542	0.594	0.619
		NTX6	-26.145	±	19.195					
	P2	sham	4.875	±	0.315	1	-2.750	0.222	0.400	1.944
		NTX6	24.895	±	6.965					
	N2	sham	-16.740	±	0.970	1	-1.829	0.318	0.359	1.294
		NTX6	-7.410	±	4.130					
	P3	sham	8.475	±	4.915	1	-0.969	0.510	0.574	0.685
		NTX6	19.530	±	6.490					

SEM: standard error of the mean, df: degrees of freedom related to available channel data, FDR-*p*: false discovery rate-adjusted *p*-value, $|d|$: effect size Cohen´s *d* with $|d| \geq 0.2$ = small, $|d| \geq 0.5$ = medium and $|d| > 0.8$ = large effects. Significant *p*-values given in bold, italic

Table S14 Statistical analysis of ERP latencies following NTX administration at 30 µg/µl

Electrode	ERP component	Comparison	Latency mean ± SEM (ms)			df	*t*	*p*	FDR-*p*	|*d*|
PR	P1	sham	60.934	±	1.679	4	2.549	0.063	0.186	1.140
		NTX30	53.266	±	1.678					
	N1	sham	96.268	±	4.527	4	3.504	***0.025***	0.065	1.567
		NTX30	87.000	±	2.974					
	P2	sham	122.000	±	3.000	4	0.261	0.669	0.903	0.206
		NTX30	119.534	±	3.374					
	N2	sham	143.066	±	5.017	4	-0.091	0.932	0.932	0.041
		NTX30	143.932	±	8.545					
	P3	sham	296.666	±	35.647	4	-0.833	0.451	0.822	0.373
		NTX30	365.600	±	52.279					
MR	P1	sham	61.918	±	5.465	3	1.268	0.294	0.441	0.634
		NTX30	53.583	±	1.859					
	N1	sham	104.333	±	0.667	3	4.572	***0.020***	0.065	2.286
		NTX30	89.168	±	3.169					
	P2	sham	121.250	±	3.750	3	0.000	1.000	1.000	0.329
		NTX30	121.250	±	3.750					
	N2	sham	148.500	±	8.740	3	0.712	0.528	0.792	0.356
		NTX30	138.333	±	8.333					
	P3	sham	334.165	±	36.492	3	0.698	0.536	0.822	0.349
		NTX30	281.415	±	42.846					
FR	P1	sham	66.585	±	2.579	3	2.567	0.083	0.186	1.283
		NTX30	49.668	±	5.712					
	N1	sham	98.668	±	2.241	3	2.545	0.084	0.108	1.272
		NTX30	91.500	±	1.664					
	P2	sham	117.250	±	3.092	3	-2.506	0.087	0.783	1.253
		NTX30	125.000	±	0.000					
	N2	sham	137.583	±	7.583	3	-1.731	0.182	0.792	0.865
		NTX30	140.833	±	8.849					
	P3	sham	315.833	±	21.008	3	-3.490	***0.040***	0.358	1.745
		NTX30	415.833	±	46.114					
FC	P1	sham	61.134	±	6.763	4	0.858	0.439	0.496	0.384
		NTX30	54.798	±	1.068					
	N1	sham	99.666	±	2.474	4	2.602	0.060	0.108	1.163
		NTX30	90.000	±	1.650					
	P2	sham	121.600	±	3.400	4	-0.523	0.629	0.880	0.234
		NTX30	123.666	±	1.334					
	N2	sham	140.466	±	7.586	4	0.256	0.811	0.912	0.114
		NTX30	137.334	±	7.334					
	P3	sham	332.800	±	42.004	4	-0.370	0.730	0.822	0.165
		NTX30	365.066	±	53.830					
PC	P1	sham	64.915	±	1.729	3	3.641	***0.036***	0.186	1.820
		NTX30	53.668	±	1.459					
	N1	sham	100.918	±	3.074	3	5.484	***0.012***	0.065	2.742
		NTX30	89.333	±	3.683					
	P2	sham	118.918	±	3.698	3	0.034	0.975	1.000	0.017
		NTX30	118.668	±	3.778					
	N2	sham	138.668	±	5.308	3	-1.732	0.182	0.792	0.866
		NTX30	146.583	±	9.724					
	P3	sham	302.918	±	39.653	3	-1.864	0.159	0.716	0.932
		NTX30	389.500	±	37.990					

Table S14 Statistical analysis of ERP latencies following NTX administration at 30 µg/µl (continued)

Electrode	ERP component	Comparison	Latency mean ± SEM (ms)			df	*t*	*p*	FDR-*p*	|*d*|
MC	P1	sham	59.665	±	5.130	3	0.886	0.441	0.496	0.443
		NTX30	55.500	±	1.456					
	N1	sham	93.500	±	5.158	3	0.519	0.640	0.640	0.259
		NTX30	89.830	±	3.304					
	P2	sham	123.333	±	1.668	3	0.421	0.702	0.903	0.211
		NTX30	121.500	±	3.500					
	N2	sham	151.915	±	8.643	3	0.384	0.727	0.912	0.192
		NTX30	147.833	±	10.318					
	P3	sham	328.335	±	43.903	3	-0.188	0.863	0.863	0.094
		NTX30	345.000	±	53.589					
FL	P1	sham	59.998	±	6.887	4	0.719	0.512	0.512	0.321
		NTX30	54.534	±	1.157					
	N1	sham	100.534	±	2.370	4	3.333	***0.029***	0.065	1.490
		NTX30	89.934	±	1.270					
	P2	sham	122.000	±	3.000	4	-1.000	0.374	0.842	0.447
		NTX30	125.000	±	0.000					
	N2	sham	147.400	±	8.009	4	0.841	0.448	0.792	0.376
		NTX30	137.800	±	7.800					
	P3	sham	311.934	±	39.098	4	-0.772	0.483	0.822	0.345
		NTX30	371.266	±	47.701					
ML	P1	sham	64.532	±	3.170	4	2.574	0.062	0.186	1.151
		NTX30	54.200	±	1.396					
	N1	sham	100.532	±	2.616	4	1.938	0.125	0.140	0.867
		NTX30	90.532	±	2.895					
	P2	sham	123.600	±	1.400	4	-1.000	0.374	0.842	0.447
		NTX30	125.000	±	0.000					
	N2	sham	150.068	±	8.275	4	0.756	0.492	0.792	0.338
		NTX30	145.066	±	9.227					
	P3	sham	326.268	±	33.821	4	-0.443	0.680	0.822	0.198
		NTX30	358.734	±	45.044					
PL	P1	sham	60.253	±	2.790	3	1.379	0.262	0.441	0.689
		NTX30	50.080	±	4.679					
	N1	sham	100.665	±	3.799	3	2.623	0.079	0.108	1.311
		NTX30	85.500	±	3.634					
	P2	sham	125.000	±	0.000	3	1.539	0.221	0.842	0.770
		NTX30	119.750	±	3.411					
	N2	sham	147.833	±	10.340	3	1.197	0.317	0.792	0.599
		NTX30	138.250	±	8.250					
	P3	sham	315.750	±	41.082	3	0.548	0.622	0.822	0.274
		NTX30	277.500	±	42.430					

SEM: standard error of the mean, df: degrees of freedom related to available channel data, FDR-*p*: false discovery rate-adjusted *p*-value, $|d|$: effect size Cohen´s *d* with $|d| \geq 0.2$ = small, $|d| \geq 0.5$ = medium and $|d| > 0.8$ = large effects. Significant *p*-values given in bold, italic

Table S15 Statistical analysis of ERP amplitudes following NTX administration at 30 µg/µl

Electrode	ERP component	Comparison	Amplitude mean ± SEM (µV)			df	*t*	*p*	FDR-*p*	$\lvert d \rvert$
PR	P1	sham	4.484	±	3.788	4	-2.158	0.097	0.197	0.965
		NTX30	20.818	±	5.443					
	N1	sham	-10.160	±	4.848	4	1.463	0.217	0.217	0.654
		NTX30	-36.068	±	15.921					
	P2	sham	6.684	±	2.511	4	-1.630	0.178	0.216	0.729
		NTX30	27.016	±	11.180					
	N2	sham	-5.698	±	4.714	4	0.027	0.980	0.980	0.012
		NTX30	-5.864	±	5.555					
	P3	sham	10.716	±	2.480	4	-2.218	0.091	0.242	0.992
		NTX30	23.812	±	4.018					
MR	P1	sham	3.058	±	2.927	3	-1.978	0.142	0.197	0.989
		NTX30	26.343	±	11.839					
	N1	sham	-4.115	±	4.600	3	1.979	0.142	0.194	0.990
		NTX30	-33.193	±	13.087					
	P2	sham	4.863	±	1.339	3	-1.463	0.240	0.240	0.732
		NTX30	13.290	±	5.105					
	N2	sham	-8.418	±	2.921	3	2.075	0.130	0.482	1.038
		NTX30	-20.280	±	7.028					
	P3	sham	8.153	±	2.062	3	-1.875	0.157	0.242	0.938
		NTX30	20.700	±	4.817					
FR	P1	sham	7.293	±	3.203	3	-1.848	0.162	0.197	0.924
		NTX30	15.195	±	6.202					
	N1	sham	-5.380	±	1.803	3	3.924	***0.029***	0.088	1.962
		NTX30	-25.360	±	6.878					
	P2	sham	1.898	±	1.316	3	-3.277	***0.047***	0.133	1.638
		NTX30	14.668	±	5.157					
	N2	sham	-5.188	±	2.672	3	0.823	0.471	0.706	0.411
		NTX30	-9.710	±	6.922					
	P3	sham	9.093	±	1.707	3	-3.778	***0.032***	0.242	1.889
		NTX30	16.970	±	2.115					
FC	P1	sham	6.816	±	6.110	4	-1.548	0.197	0.197	0.692
		NTX30	22.478	±	7.464					
	N1	sham	-5.864	±	2.957	4	5.851	***0.004***	***0.019***	2.617
		NTX30	-30.964	±	5.809					
	P2	sham	4.390	±	3.269	4	-3.947	***0.017***	0.077	1.765
		NTX30	19.508	±	4.866					
	N2	sham	-2.900	±	6.936	4	1.004	0.372	0.670	0.449
		NTX30	-8.574	±	5.612					
	P3	sham	15.502	±	5.443	4	-0.206	0.847	0.847	0.092
		NTX30	17.010	±	3.805					
PC	P1	sham	6.688	±	3.967	3	-1.693	0.189	0.197	0.847
		NTX30	12.253	±	4.178					
	N1	sham	-6.728	±	3.591	3	1.874	0.158	0.194	0.937
		NTX30	-18.688	±	5.237					
	P2	sham	2.710	±	1.826	3	-2.532	0.085	0.153	1.266
		NTX30	12.445	±	4.698					
	N2	sham	-4.880	±	3.659	3	1.238	0.304	0.670	0.619
		NTX30	-9.243	±	1.198					
	P3	sham	12.830	±	1.539	3	-1.172	0.326	0.366	0.586
		NTX30	16.160	±	2.019					

Table S15 Statistical analysis of ERP amplitudes following NTX administration at 30 µg/µl (continued)

Electrode	ERP component	Comparison	Amplitude mean ± SEM (µV)			df	*t*	*p*	FDR-*p*	|*d*|
MC	P1	sham	2.543	±	4.117	3	-1.826	0.165	0.197	0.913
		NTX30	25.553	±	9.365					
	N1	sham	-4.938	±	2.002	3	3.502	***0.039***	0.089	1.751
		NTX30	-25.923	±	7.325					
	P2	sham	4.840	±	1.768	3	-2.980	0.059	0.133	1.500
		NTX30	16.410	±	5.022					
	N2	sham	-4.323	±	4.856	3	1.854	0.161	0.482	0.927
		NTX30	-9.793	±	2.054					
	P3	sham	11.348	±	2.828	3	-1.562	0.216	0.278	0.781
		NTX30	24.420	±	6.335					
FL	P1	sham	4.992	±	3.581	4	-2.067	0.108	0.197	0.924
		NTX30	22.748	±	6.542					
	N1	sham	-8.520	±	1.676	4	10.436	***0.000***	***0.004***	4.667
		NTX30	-35.810	±	3.726					
	P2	sham	4.274	±	1.874	4	-6.965	***0.002***	***0.018***	3.115
		NTX30	19.958	±	2.807					
	N2	sham	-5.342	±	4.224	4	2.345	0.079	0.482	1.049
		NTX30	-13.530	±	3.675					
	P3	sham	11.868	±	1.930	4	-1.849	0.138	0.242	0.827
		NTX30	23.112	±	5.060					
ML	P1	sham	2.800	±	2.704	4	-1.965	0.121	0.197	0.879
		NTX30	25.532	±	10.116					
	N1	sham	-6.728	±	1.476	4	2.595	0.060	0.109	1.160
		NTX30	-37.256	±	12.779					
	P2	sham	3.716	±	1.089	4	-2.083	0.106	0.159	0.932
		NTX30	27.184	±	11.846					
	N2	sham	-6.776	±	2.748	4	0.140	0.896	0.980	0.062
		NTX30	-7.386	±	2.935					
	P3	sham	8.084	±	1.392	4	-1.930	0.126	0.242	0.863
		NTX30	23.830	±	7.366					
PL	P1	sham	0.965	±	2.590	3	-1.950	0.146	0.197	0.975
		NTX30	20.633	±	9.311					
	N1	sham	-9.623	±	4.840	3	1.784	0.172	0.194	0.892
		NTX30	-43.940	±	19.323					
	P2	sham	4.033	±	0.894	3	-1.676	0.192	0.216	0.838
		NTX30	30.373	±	16.145					
	N2	sham	-9.213	±	4.386	3	0.449	0.684	0.879	0.225
		NTX30	-11.328	±	1.122					
	P3	sham	9.458	±	2.092	3	-1.852	0.161	0.242	0.926
		NTX30	19.018	±	3.260					

SEM: standard error of the mean, df: degrees of freedom related to available channel data, FDR-*p*: false discovery rate-adjusted p-value, $|d|$: effect size Cohen´s *d* with $|d| \geq 0.2$ = small, $|d| \geq 0.5$ = medium and $|d| > 0.8$ = large effects. Significant *p*-values given in bold, italic

Table S16 Contingency tables of treatment classifications

		Predicted	
		sham	alcohol high
Actual	sham	8	1
	alcohol high	0	9

		Predicted	
		alcohol low	el. stim.
Actual	alcohol low	6	0
	el. stim.	0	6

		Predicted	
		sham	alcohol low
Actual	sham	4	2
	alcohol low	2	4

		Predicted	
		sham	NTX3
Actual	sham	4	0
	NTX3	1	3

		Predicted	
		alcohol low	alcohol high
Actual	alcohol low	4	2
	alcohol high	0	6

		Predicted	
		sham	NTX6
Actual	sham	4	0
	NTX6	2	2

		Predicted	
		sham	el. stim.
Actual	sham	6	2
	el. stim.	2	6

		Predicted	
		sham	NTX30
Actual	sham	5	0
	NTX30	1	4

		Predicted	
		alcohol high	el. stim.
Actual	alcohol high	7	0
	el. stim.	1	6

Table S16 Contingency tables of treatment classifications (continued)

		Predicted	
		alcohol high	NTX3
Actual	alcohol high	3	0
	NTX3	0	3

		Predicted	
		alcohol low	NTX3
Actual	alcohol low	2	1
	NTX3	1	2

		Predicted	
		alcohol high	NTX6
Actual	alcohol high	3	0
	NTX6	1	2

		Predicted	
		alcohol low	NTX6
Actual	alcohol low	2	1
	NTX6	1	2

		Predicted	
		alcohol high	NTX30
Actual	alcohol high	4	0
	NTX30	2	2

		Predicted	
		alcohol low	NTX30
Actual	alcohol low	4	0
	NTX30	2	2

Table S17 Statistical analysis of immunohistochemical brain tissue stainings

Antigene	Group	Parameter mean	±	SEM	One-way ANOVA		
		**Fluorescence intensity (× 10^3)**			***F***	***p***	***f***
NeuN	sham	38.811	±	2.356	0.029	0.971	0.076
	dummy	38.081	±	3.157			
	treatment	38.790	±	1.614			
		**%Area of ROI**					
	sham	26.572	±	1.236	2.253	0.156	0.671
	dummy	28.291	±	0.978			
	treatment	24.401	±	1.203			
		**Counts/mm²**					
	sham	1761.670	±	129.939	4.474	***0.041***	0.946
	dummy	1914.000	±	93.243			
	treatment	1607.290	±	42.912			
		**Fluorescence intensity (× 10^3)**					
GFAP	sham	12.454	±	1.569	0.605	0.565	0.348
	dummy	13.779	±	1.293			
	treatment	11.952	±	0.889			
		**%Area of ROI**					
	sham	8.683	±	2.043	0.481	0.632	0.310
	dummy	10.610	±	1.593			
	treatment	8.397	±	1.292			
		**Counts/mm²**					
	sham	1729.000	±	261.443	0.430	0.662	0.293
	dummy	1961.000	±	216.980			
	treatment	1734.430	±	129.560			
		**Fluorescence intensity (× 10^3)**					
Iba1	sham	9.437	±	0.957	0.982	0.408	0.443
	dummy	10.946	±	0.784			
	treatment	10.240	±	0.443			
		**%Area of ROI**					
	sham	3.013	±	0.755	0.433	0.660	0.294
	dummy	4.742	±	2.148			
	treatment	4.156	±	0.750			
		**Counts/mm²**					
	sham	758.667	±	135.225	0.433	0.660	0.294
	dummy	1040.000	±	385.509			
	treatment	1013.570	±	142.863			
		**Fluorescence intensity (× 10^3)**					
Caspase3	sham	3.927	±	0.121	0.221	0.806	0.210
	dummy	5.114	±	0.419			
	treatment	5.051	±	1.267			
		**%Area of ROI**					
	sham	0.780	±	0.149	0.004	0.996	0.027
	dummy	0.748	±	0.288			
	treatment	0.755	±	0.211			
		**Counts/mm²**					
	sham	510.000	±	105.671	0.647	0.544	0.360
	dummy	358.000	±	109.929			
	treatment	411.290	±	57.943			
		**Fluorescence intensity (× 10^3)**					
Laminin	sham	28.076	±	2.428	8.793	***0.006***	1.326
	dummy	28.092	±	2.482			
	treatment	20.331	±	0.953			
		**%Area of ROI**					
	sham	18.699	±	2.032	7.853	***0.009***	1.253
	dummy	19.556	±	2.018			
	treatment	12.703	±	0.931			
		**Counts/mm²**					
	sham	1692.330	±	68.635	1.051	0.385	0.458
	dummy	1769.670	±	121.469			
	treatment	1577.860	±	82.591			
		**Fluorescence intensity (× 10^3)**					
CD31	sham	6.070	±	0.700	1.270	0.322	0.504
	dummy	6.542	±	1.024			
	treatment	8.140	±	0.911			
		**%Area of ROI**					
	sham	5.531	±	0.807	0.142	0.870	0.168
	dummy	6.253	±	1.444			
	treatment	5.767	±	0.556			
		**Counts/mm²**					
	sham	2306.667	±	72.880	3.673	0.064	0.857
	dummy	2395.330	±	273.980			
	treatment	1814.290	±	138.067			

SEM: standard error of the mean, *f*: effect size Cohen´s *f* with $f \geq 0.1$ = small, $f \geq 0.25$ = medium and $f > 0.4$ = large effects. Significant *p*-values given in bold, italic
